# Bispecific antibodies in gastrointestinal cancers: current applications and future perspectives

**DOI:** 10.3389/fonc.2026.1927829

**Published:** 2026-09-09

**Authors:** Mariam Ismail, Noran Al-Gizey, Ebtesam Al-Najjar, Zaid Alabed, Nour Mustafa, Yazan Hamdaneh, Abdullah Esmail

**Affiliations:** 1 Faculty of Medicine, Delta University for Science and Technology, Gamasa, Egypt; 2 College of Medicine, University of Baghdad, Baghdad, Iraq; 3 Houston Methodist Cancer Center, Houston Methodist Hospital, Houston, TX, United States; 4 Faculty of Medicine, the University of Jordan, Amman, Jordan; 5 Department of Medicine, Houston Methodist Cancer Center, Houston Methodist Hospital, Houston, TX, United States; 6 Department of Medicine, Weill Cornell Medical College, New York, NY, United States

**Keywords:** bispecific antibodies, cancers, gastrointestinal, HER2, zanidatamab

## Abstract

Gastrointestinal (GI) cancers remain a leading cause of cancer-related morbidity and mortality worldwide despite major advances in systemic therapy. Although immune checkpoint inhibitors have improved outcomes in selected biomarker-defined populations, most GI malignancies, including microsatellite-stable colorectal cancer, pancreatic ductal adenocarcinoma, and many biliary tract tumors, remain largely resistant because of low tumor immunogenicity, stromal exclusion, immune suppression, and adaptive resistance mechanisms. Bispecific antibodies (BsAbs) have emerged as a rapidly expanding class of immunotherapeutics capable of simultaneously engaging two distinct targets, enabling mechanisms such as T-cell redirection, dual checkpoint inhibition, combined immune and anti-angiogenic modulation, tumor-selective co-stimulation, and stromal remodeling. Recent clinical advances, including the approval of HER2-targeted zanidatamab and the emergence of multiple late-phase studies across GI malignancies, have validated the therapeutic potential of this platform. This review examines the biological rationale, structural formats, and mechanisms of action of BsAbs and summarizes current clinical evidence across colorectal, gastric, gastroesophageal, hepatocellular, pancreatic, biliary tract, esophageal, and neuroendocrine cancers. We also discuss emerging therapeutic targets, biomarker-driven patient selection, safety considerations, combination strategies, manufacturing challenges, and future directions. Collectively, current evidence suggests that BsAbs are poised to become an increasingly important component of precision immunotherapy, with the potential to overcome key limitations of conventional immunotherapeutic approaches in GI oncology.

## 1 Introduction

Gastrointestinal (GI) cancers comprise a diverse group of malignancies arising from the esophagus, stomach, colorectum, liver, pancreas, and biliary tract and remain a major global cause of cancer-related morbidity and mortality. In 2023, colorectal cancer was the third most commonly diagnosed cancer worldwide, accounting for 2.29 million new cases and 1.11 million deaths, while gastric cancer accounted for 1.26 million cases and 935,000 deaths, and esophageal cancer caused 577,000 deaths (1). Collectively, GI malignancies account for approximately 26% of global cancer incidence and 35% of cancer-related mortality, with projections suggesting that the annual burden could exceed 9 million new cases and 6 million deaths by 2050 (2, 3).

The introduction of immune checkpoint inhibitors (ICIs) targeting the PD-1/PD-L1 and CTLA-4 pathways has transformed the management of several GI malignancies. Regulatory approvals now extend across multiple disease settings, including microsatellite instability-high (MSI-H) colorectal cancer, PD-L1-positive gastroesophageal adenocarcinoma, hepatocellular carcinoma, esophageal squamous cell carcinoma, and biliary tract cancer (4, 5). However, the clinical benefit of checkpoint blockade remains highly heterogeneous. Durable responses are largely confined to biomarker-selected populations, whereas most patients, including those with microsatellite-stable (MSS) colorectal cancer, pancreatic ductal adenocarcinoma, and many biliary tract cancers, derive limited benefit from current immunotherapeutic approaches (6, 7). This reflects the complex biology of GI tumors, which is characterized by low immunogenicity, impaired T-cell infiltration, stromal exclusion, adaptive immune resistance, and profound immunosuppressive tumor microenvironments enriched with regulatory T cells, myeloid-derived suppressor cells, and tumor-associated macrophages (5).

Bispecific antibodies (BsAbs) have emerged as a versatile immunotherapeutic platform capable of addressing several of these resistance mechanisms simultaneously. By engaging two distinct antigens or signaling pathways within a single molecule (8, 9), BsAbs can redirect cytotoxic immune cells toward tumor cells, block complementary immune checkpoints, combine immune modulation with anti-angiogenic activity, enhance co-stimulatory signaling, or selectively remodel the tumor microenvironment (8, 9). The field has expanded rapidly over the past decade, with 14 BsAbs receiving regulatory approval globally, including 11 for oncologic indications, and more than 680 registered clinical trials evaluating 183 agents in solid tumors (8, 10). GI malignancies represent the largest area of investigation, accounting for more than one-fifth of all registered solid-tumor BsAb studies (10).

Clinical development in GI oncology has accelerated considerably in recent years. Zanidatamab, a biparatopic HER2-directed bispecific antibody, became the first BsAb approved for a GI malignancy following its accelerated approval for previously treated HER2-positive biliary tract cancer. Subsequent phase III data demonstrated significant survival benefits for zanidatamab-based regimens in HER2-positive gastroesophageal adenocarcinoma (11). At the same time, dual-checkpoint bispecific antibodies such as cadonilimab, PD-1/VEGF-targeting constructs, and DLL3-directed T-cell engagers have generated encouraging results across gastric, colorectal, hepatobiliary, and neuroendocrine cancers, highlighting the broad therapeutic potential of this platform (**Table 1**).

**Table 1:** Selected bispecific antibodies in clinical development for colorectal cancer.

Agent	Target(s)	Setting	Key findings	Development stage	References
Cibisatamab	CEA×CD3	MSS mCRC/CEA-positive solid tumors	Improved activity with atezolizumab or FAP-4-1BBL; increased CD8+ T-cell infiltration	Phase 1/1b	(69, 70)
CBI-1214	LY6G6D×CD3	MSS/MSI-L CRC	First-in-human evaluation after promising preclinical activity at low antigen density	Phase 1	(106)
Zanidatamab	Biparatopic HER2	HER2-positive CRC	Responses observed including prior HER2-treated patients	Phase 1/2	(82)
KN026 + KN046	HER2 + PD-L1/CTLA-4	HER2-positive CRC	High response rate and prolonged PFS in heavily pretreated disease	Phase 2	(86)
Amivantamab	EGFR×MET	RAS/BRAF WT mCRC	Activity in anti-EGFR-resistant disease and across CMS2/CMS4 subtypes	Phase 1b/2; Phase 3 ongoing	(103, 107–109)
INCA33890	PD-1×TGFβR2	MSS mCRC	Activity including patients with liver metastases; phase 3 initiated	Phase 1; Phase 3 ongoing	(110)
Ivonescimab	PD-1×VEGF	First-line mCRC	High response rates with mFOLFOX6; phase 3 ongoing	Phase 2; Phase 3 ongoing	(111, 114)
PF-08634404	PD-1×VEGF	First-line mCRC	Phase 3 evaluation with mFOLFOX6	Phase 3	(113)
Pumitamig	PD-L1×VEGF-A	First-line mCRC	Being evaluated with chemotherapy	Phase 2/3	(112)
DSP107 + atezolizumab	CD47×4-1BBL + PD-L1	MSS mCRC	Durable responses and encouraging disease control	Phase 2	(115)
BNT314 + pumitamig	EpCAM×4-1BB + PD-L1×VEGF-A	MSS mCRC	Early multi-bispecific combination strategy with chemotherapy	Phase 1/2	(116)

The growing diversity of BsAb architecture reflects the biological heterogeneity of GI malignancies. Current approaches include T-cell-engaging antibodies directed against tumor-associated antigens such as CEA, CLDN18.2, mesothelin, GPC3, DLL3, and HER2; biparatopic antibodies designed to enhance receptor blockade; dual immune-checkpoint inhibitors targeting pathways including PD-1, CTLA-4, TIGIT, VEGF, and TGF-β; and co-stimulatory constructs that promote localized immune activation within the tumor microenvironment (10, 12). Together, these strategies seek not only to enhance antitumor immunity but also to overcome the multifactorial mechanisms that limit the effectiveness of conventional immunotherapy in GI cancers (**Table 2**).

**Table 2:** Selected CLDN18.2-directed bispecific antibody platforms in gastric and gastroesophageal junction cancers.

Agent	Target(s)	Platform	Development stage	Population/Model	Key findings	Key safety findings	References
AMG 910	CLDN18.2×CD3	Half-life extended BiTE	Preclinical	GSU and SNU-620 gastric cancer xenografts	Potent cytotoxicity with Treg reprogramming and enhanced effector T-cell signaling	Cytokine release observed preclinically	(144)
AZD5863	CLDN18.2×CD3	Affinity-modulated T-cell engager	Preclinical	Gastric, pancreatic, and esophageal cancer models	Potent antitumor activity with reduced cytokine release and improved therapeutic index	Reduced cytokine release relative to conventional CD3 engagers	(146)
CLDN18.2/CD3 constructs	CLDN18.2×CD3	Bispecific antibody and diabody formats	Preclinical	Gastric and pancreatic cancer models	Potent T-cell-dependent cytotoxicity and tumor inhibition in PDX models	No obvious gastric toxicity in murine studies	(145)
IBI389	CLDN18.2×CD3	T-cell engager	Phase I	Advanced gastric/GEJ cancers	ORR 22.2%; DCR 74.1%; median PFS 4.3 months; median OS 10.3 months	CRS predominantly grade 1-2; no treatment-related deaths	(126)
QLS31905 + chemotherapy	CLDN18.2×CD3	T-cell engager + chemotherapy	Phase Ib/II	First-line CLDN18.2-positive gastric cancer	ORR 74.4%; DCR 93.0%; activity maintained in low-expression tumors	Manageable toxicity profile	(147)
Q-1802 + XELOX	CLDN18.2×PD-L1	Checkpoint-integrated bispecific antibody	Phase I/II	Treatment-naive CLDN18.2-positive GC/GEJ	ORR 70.0%; median PFS 11.3 months; ORR 81.8% in CLDN18.2-high/PD-L1 CPS ≥5 subgroup	No treatment-related deaths; manageable toxicity	(148)
Givastomig	CLDN18.2×4-1BB	Conditional co-stimulatory bispecific antibody	Phase I	CLDN18.2-positive advanced solid tumors	Preliminary antitumor activity in gastroesophageal cancer	No dose-limiting toxicities reported	(149)
PM1032	CLDN18.2×4-1BB	Co-stimulatory bispecific antibody	Phase I/II	Advanced solid tumors	Durable disease control observed in selected CLDN18.2-positive gastric/GEJ patients	Acceptable safety profile	(150)

ADCC, antibody-dependent cellular cytotoxicity; BiTE, bispecific T-cell engager; CLDN18.2, claudin 18.2; CPS, combined positive score; CRS, cytokine release syndrome; DCR, disease control rate; GEJ, gastroesophageal junction; ORR, objective response rate; OS, overall survival; PDX, patient-derived xenograft; PFS, progression-free survival; PD-L1, programmed death-ligand 1; Treg, regulatory T cell.

This review synthesizes current evidence on the development of bispecific antibodies across gastrointestinal malignancies, focusing on their biological rationale, therapeutic targets, clinical activity, safety profiles, and future directions.

## 2 Understanding bispecific antibodies

### 2.1 Definition, history, and structural platforms

Bispecific antibodies (BsAbs) are engineered antibody-based molecules capable of simultaneously recognizing two distinct antigens or epitopes within a single construct (8). Unlike conventional monoclonal antibodies that bind a single target, BsAbs mediate coordinated interactions between tumor cells, immune cells, stromal components, and immune signaling pathways, enabling therapeutic effects not achievable with monospecific agents (9).

The concept dates to the 1960s, when the first bispecific antigen-binding molecules were generated through chemical conjugation of two Fab fragments (13). Advances in hybridoma and quadroma technologies during the 1980s enabled monoclonal BsAb production, but early clinical development was limited by manufacturing complexity, structural instability, immunogenicity, and short serum half-life (14). Modern recombinant engineering technologies, including knobs-into-holes platforms, CrossMAb designs, dual-affinity retargeting constructs, controlled Fab-arm exchange, and asymmetric IgG-like formats, have substantially improved antibody assembly, specificity, stability, pharmacokinetics, and manufacturability (14, 15). These advances have accelerated clinical development, with 14 BsAbs now approved globally and an expanding pipeline in solid tumors, including gastrointestinal cancers (8, 16) (**Table 3**).

**Table 3:** Safety profiles of representative bispecific antibodies in gastrointestinal malignancies.

Agent (target)	Format	CRS (any/≥G3)	Key toxicities	Reference
Cibisatamab (CEA×CD3)	CD3 TCE	57.7%/3.8%	Colitis, ADA formation	(69, 70)
IBI389 (CLDN18.2×CD3)	CD3 TCE	59.5%/0.8%	GGT elevation, lymphocytopenia	(126)
Givastomig (CLDN18.2×4-1BB)	Co-stimulatory	None reported	Nausea, fatigue, ALT increase	(149)
BGB-B167 (CEA×4-1BB)	Co-stimulatory	None reported	Mild immune-related toxicities	(177)
BGB-B2033 (GPC3×4-1BB)	Co-stimulatory	None reported	ALT/AST/bilirubin elevation	(184)
Zanidatamab (HER2)	Biparatopic	None	Diarrhea, infusion reactions, reversible LVEF decline	(82, 83, 133)
Cadonilimab (PD-1×CTLA-4)	Checkpoint	None	Immune-related hepatitis, colitis, myocarditis	(64, 182, 185)
Ivonescimab (PD-1×VEGF)	Checkpoint/Anti-angiogenic	None	Proteinuria, hypertension, transaminase elevation	(159)
Q-1802 (CLDN18.2×PD-L1)	Checkpoint bispecific	None reported	Thrombocytopenia, neutropenia, anemia	(148)
Catumaxomab (EpCAM×CD3)	Trifunctional	Present	Hepatotoxicity, abdominal pain, nausea/vomiting	(183, 186)

Representative toxicity profiles of bispecific antibodies under investigation in gastrointestinal malignancies. Toxicity patterns vary according to antibody format and mechanism of action. CD3-engaging T-cell engagers are predominantly associated with cytokine release syndrome and on-target/off-tumor toxicity, whereas checkpoint- and angiogenesis-based bispecific antibodies generally exhibit adverse-event profiles like those of their parent monoclonal antibodies. ADA, anti-drug antibody; ALT, alanine aminotransferase; AST, aspartate aminotransferase; CRS, cytokine release syndrome; GGT, gamma-glutamyl transferase; LVEF, left ventricular ejection fraction.

#### 2.1.1 Current BsAb platforms are broadly divided into two categories

IgG-like bispecific antibodies retain an Fc region that provides structural stability, prolonged serum half-life through neonatal Fc receptor-mediated recycling, and preserved immune effector functions such as antibody-dependent cellular cytotoxicity and complement activation (17). Fc silencing is increasingly incorporated to reduce off-target immune activation (18). Several engineering approaches have been developed to address chain-mispairing during antibody production. Knobs-into-holes, CrossMAb, and DuoBody technologies improve heavy- and light-chain pairing, enabling efficient production of highly pure bispecific antibodies (19–21). Because of their favorable pharmacokinetic properties, IgG-like architectures are used by many clinically advanced BsAbs in GI oncology, including zanidatamab and bintrafusp alfa.

Non-IgG-like bispecific antibodies lack a conventional Fc domain and are typically composed of smaller recombinant fragments, including bispecific T-cell engagers (BiTEs), tandem single-chain variable fragments, diabodies, dual-affinity retargeting molecules, and single-domain antibody constructs (17) (**Tables 4**–**7**). Their smaller molecular size often improves tumor penetration and immune synapse formation, but the absence of an Fc region results in rapid renal clearance and shorter half-life, frequently requiring continuous infusion or repeated dosing. BiTEs are the most clinically established non-IgG-like format; blinatumomab, the first FDA-approved BiTE targeting CD19 and CD3, established the clinical feasibility of T-cell-engaging bispecific immunotherapy (22). More recently, trispecific antibodies and multifunctional immune engagers have emerged as next-generation platforms designed to target multiple tumor and immune pathways simultaneously (23).

**Table 4:** Bispecific and multispecific antibody platforms under clinical development for hepatocellular carcinoma.

Agent	Target(s)	Platform	Stage	Key findings	References
BGB-B2033	GPC3 × 4-1BB	Co-stimulatory BsAb	Phase 1	ORR 20.3% (28.9% at higher doses); durable responses; well tolerated	(184)
SAR444200	GPC3 × TCRαβ	NANOBODY-based TCE	Phase 1/2	Stable disease in 30.3%; AFP reduction in 27% of HCC patients with elevated AFP	(208, 209)
AZD9793	GPC3 × CD8 × TCR	CD8-guided trispecific TCE	Phase 1/2	First-in-human study enrolling; CD8-biased engagement with reduced cytokine release	(210)
MTS105	GPC3 × CD3 (mRNA-LNP)	Organ-specific mRNA-encoded TCE	Phase 1 (ongoing)	Complete regression in orthotopic HCC models; favorable NHP pharmacokinetics	(211)
Nilvanstomig (ZG005) + bevacizumab	PD-1 × TIGIT	Dual checkpoint BsAb	Phase 2 (randomized)	Higher ORR and prolonged PFS versus sintilimab plus bevacizumab	(216)
Ivonescimab + HAIC	PD-1 × VEGF	Checkpoint/anti-angiogenic BsAb	Phase 2	ORR 41.7% (RECIST v1.1); surgical conversion rate 58.3%	(217)
GPC3/CD47 bispecific antibody	GPC3 × CD47	Innate immune checkpoint BsAb	Preclinical	Superior activity to monotherapies and combination antibodies; macrophage- and neutrophil-dependent efficacy	(214)
GPC3/PD-L1 bispecific antibody	GPC3 × PD-L1	Tumor-directed checkpoint BsAb	Preclinical	GPC3-dependent PD-L1 blockade with preferential tumor accumulation	(213)
GPC3/CD16A bispecific antibody	GPC3 × CD16A	NK cell-redirecting BsAb	Preclinical	Potent NK-mediated cytotoxicity with synergy observed in combination with sorafenib	(215)

**Table 5:** Selected bispecific antibody platforms under investigation in pancreatic cancer.

Agent	Target(s)	Platform	Stage	Key findings	References
Spevatamig (PT886) + GnP	CLDN18.2 × CD47	Innate immune checkpoint bispecific antibody	Phase 1/2	ORR 48%; DCR 90%; median PFS 7.3 months; median OS >13 months; activity observed irrespective of CLDN18.2 expression level	(242, 243)
JNJ-79032421	Membrane-restricted MSLN × CD3	Membrane-restricted T-cell engager	Phase 1	Activity unaffected by shed mesothelin; dose-dependent T-cell infiltration and activation in xenograft models	(28)
ZW171	MSLN × CD3	Trivalent T-cell engager	Phase 1	First-in-human clinical evaluation ongoing (NCT06523803)	(235)
HPN536	MSLN × CD3 × albumin	Trispecific T-cell-activating construct	Phase 1	Potent redirected cytotoxicity and favorable pharmacokinetics supporting weekly dosing	(232)
NAV-003	MSLN × CD3ϵ	Divalent bispecific antibody	Preclinical	Enhanced activity against immunosuppressive tumors and efficacy in patient-derived xenograft models	(233)
NM28-2746 ± NM21-1480	MSLN × CD3 ± PD-L1 × 4-1BB	Bivalent T-cell engager ± co-stimulatory bispecific antibody	Preclinical	Preferential targeting of high-MSLN tumors; enhanced efficacy when combined with PD-L1/4-1BB co-stimulation	(234)
AMG 910	CLDN18.2 × CD3	Half-life extended BiTE	Preclinical	Potent activity in PDAC models; regulatory T-cell reprogramming; enhanced efficacy with PD-1 blockade	(134)
CC-3	B7-H3 × CD3	Affinity-optimized T-cell engager	Preclinical	Strong antitumor activity with reduced cytokine release through CD3 affinity tuning	(156)
FAP-CD40	FAP × CD40	Tumor-directed co-stimulatory bispecific antibody	Preclinical	Complete regression in KPC models; strictly FAP-dependent activation with favorable safety profile	(248, 249)
EGFR×HER2 T-BsAb	EGFR × HER2 × CD3	Heterodimeric T-cell engager	Preclinical	Potent activity in EGFR+/HER2+ cell-line-derived and patient-derived xenograft models	(245)
PD-L1/CXCR4 nanobody	PD-L1 × CXCR4	Bispecific nanobody	Preclinical	Superior antitumor efficacy compared with atezolizumab; enhanced T-cell infiltration	(246)

**Table 6:** Selected bispecific antibodies in clinical development for biliary tract cancer.

Agent	Target(s)	Setting	Key findings	Stage	References
Zanidatamab	Biparatopic HER2	Previously treated HER2-positive BTC	cORR 51.6%, mDOR 14.9 months, mOS 18.1 months in IHC 3+ tumors	FDA approved; phase III ongoing	(260, 264)
Bintrafusp alfa	PD-L1/TGF-β	First-line and later-line BTC	Phase I ORR 20%; phase II ORR 10.7%; phase II/III first-line trial showed no benefit over GemCis	First-line program terminated	(265, 266, 287)
Cadonilimab + GemCis	PD-1×CTLA-4	First-line advanced BTC	ORR 59.3%; DCR 88.1%; mPFS 9.0 months	Phase II	(270)
Cadonilimab + GemCis	PD-1×CTLA-4	Conversion therapy for unresectable BTC	ORR 54.5%; R0 conversion 18.2% in treatment-naive patients	Phase II	(271)
Ivonescimab + GemCis	PD-1×VEGF	First-line advanced BTC	ORR 63.6%; DCR 100%; mPFS 8.5 months; mOS 16.8 months	Phase II	(273)
Ivonescimab + GemCis + SBRT	PD-1×VEGF	Advanced BTC	ORR 55.0%; DCR 90.0%	Phase II	(274)
Tovecimig (CTX-009) + paclitaxel	DLL4×VEGF-A	Second-line advanced BTC	ORR 37.5% in phase II; randomized phase II/III ongoing	Phase II/III	(275)
Vudalimab	PD-1×CTLA-4	Previously treated advanced BTC	Phase II enrolling	Phase II	(269)
Cadonilimab + LM-302	PD-1×CTLA-4 + CLDN18.2 ADC	CLDN18.2-positive BTC after chemotherapy and ICI	Best overall response 50% in dose escalation	Phase Ib/II	(281)
Cabotamig (ARB202)	CDH17×CD3	CDH17-positive advanced GI cancers including CCA	Dose-responsive biomarker activity; manageable CRS	Phase I	(285)
FGFR2 biparatopic antibodies	Biparatopic FGFR2	FGFR2 fusion-driven CCA	*In vivo* efficacy, synergy with FGFR inhibitors, activity against kinase-domain resistance mutations	Preclinical	(282)

**Table 7:** Selected bispecific antibodies in clinical development for esophageal cancer and gastrointestinal neuroendocrine neoplasms.

Agent	Target(s)	Disease	Key findings	Stage	References
Cadonilimab + taxane/cisplatin	PD-1×CTLA-4	First-line ESCC	ORR 81.4%; DCR 97.7%; mPFS 7.10 months	Phase 2	(297)
Cadonilimab monotherapy	PD-1×CTLA-4	Pretreated ESCC	ORR 18.2%; mPFS 3.5 months; mOS 9.4 months	Phase 1b/2	(64)
KN046 + chemotherapy + RT	PD-L1×CTLA-4	Advanced ESCC	ORR 41.7%; mPFS 7.8 months; mOS 15.9 months	Phase 1b	(299)
SHR-1701 + neoadjuvant CRT	PD-L1/TGF-βRII	Resectable LA-ESCC	pCR 30.0%; R0 resection 100%	Phase 2	(300)
BL-B01D1	EGFR/HER3 BsAb-ADC	Pretreated ESCC	cORR 39.6% at RP2D; DCR 79.2%	Phase 1b; Phase 3 initiated	(308)
IBI334	EGFR/B7-H3	Advanced solid tumors including esophageal cancer	Enhanced EGFR targeting with favorable safety profile	Phase 1	(301)
Obrixtamig (BI 764532)	DLL3×CD3	DLL3-high epNEC (including GEP-NEC)	ORR 40.0%; ORR 50.0% in GEP-NEC; mDoR 7.9 months	Phase 1; Phase 2 enrolling	(303, 304)
Alveltamig (ZG006)	DLL3/DLL3×CD3	Refractory NEC including GI-NEC	ORR 56.3% in DLL3-high tumors; mPFS 8.41 months	Phase 2	(305)
Peluntamig (PT217)	DLL3×CD47	DLL3-positive cancers including GEP-NEC	Monotherapy and combination cohorts ongoing	Phase 1/2	(306)
Tarlatamab	DLL3×CD3	DLL3-positive tumors	Basket trial enrolling all DLL3-positive tumor types	Phase 2	(266)

### 2.2 Mechanisms of action

BsAbs exert their therapeutic effects through four principal mechanisms, each addressing distinct aspects of antitumor immunity.

#### 2.2.1 T-cell engagement

T-cell-engaging bispecific antibodies (TCEs) simultaneously bind a tumor-associated antigen on malignant cells and CD3 on cytotoxic T lymphocytes, physically redirecting T cells toward tumor cells independent of native T-cell receptor specificity or major histocompatibility complex restriction (9). This interaction promotes immune synapse formation, T-cell activation, and tumor-cell lysis through perforin and granzyme release. Unlike checkpoint inhibitors, which rely on pre-existing antitumor immunity, TCEs can activate polyclonal T-cell populations regardless of baseline tumor immunogenicity, a feature particularly relevant in immune-cold GI malignancies such as microsatellite-stable colorectal cancer and pancreatic ductal adenocarcinoma (12).

#### 2.2.2 Dual immune checkpoint blockade

Many GI tumors develop adaptive resistance through upregulation of multiple inhibitory receptors. BsAbs targeting dual checkpoint pathways aim to overcome these compensatory mechanisms by blocking two inhibitory signals simultaneously. Current approaches include PD-1/CTLA-4 (cadonilimab), PD-L1/TGF-β (bintrafusp alfa), PD-1/VEGF (ivonescimab), PD-1/LAG-3 (tebotelimab), PD-1/TIGIT (rilvegostomig), and PD-1/TIM-3 (AZD7789) combinations (10, 24).

#### 2.2.3 Tumor microenvironment modulation

Dense stromal desmoplasia, cancer-associated fibroblasts, abnormal vasculature, and immunosuppressive immune-cell populations collectively impair immune-cell infiltration in many GI cancers. BsAbs designed to modulate the tumor microenvironment include fibroblast activation protein (FAP)-targeted co-stimulatory constructs such as FAP-4-1BBL (RO7122290), which induces localized T-cell co-stimulation within FAP-expressing tumor stroma while minimizing systemic immune activation and the hepatotoxicity previously observed with systemic 4-1BB agonists (25). Similarly, FAP-CD40 bispecific antibodies provide strictly tumor-localized CD40 stimulation, achieving complete tumor regression in preclinical pancreatic cancer models without systemic toxicity.

#### 2.2.4 Improving tumor specificity

Low-level expression of tumor-associated antigens in normal tissues can lead to on-target/off-tumor toxicity and excessive cytokine release. Several engineering strategies address this challenge. Affinity tuning of CD3 binding preserves antitumor activity while reducing cytokine release (26). Membrane-proximal epitope targeting enhances immune synapse formation and overcomes interference from soluble shed antigens, as demonstrated with mesothelin-directed constructs in pancreatic cancer (27, 28). Conditional activation approaches, including masked antibodies, protease-activatable constructs, and tumor-restricted co-stimulatory molecules, further enhance tumor selectivity by restricting antibody activity to the tumor microenvironment (10).

## 3 Biological rationale for bispecific antibodies in gastrointestinal oncology

### 3.1 The immune landscape of GI tumors

Immune checkpoint inhibitors have transformed the treatment of several malignancies, but responses across GI cancers remain highly heterogeneous. Durable benefit is largely confined to selected subgroups, including MSI-H/dMMR colorectal cancer, PD-L1-positive gastroesophageal tumors, and a subset of hepatocellular carcinomas (29, 30). The majority of GI malignancies derive limited benefit from current immunotherapy because of overlapping biological barriers that collectively restrict effective antitumor immunity (5) (**Tables 4**–**7**).

Tumor immunogenicity varies substantially across GI cancer subtypes. MSI-H/dMMR tumors harbor high tumor mutational burdens and generate abundant neoantigens, resulting in dense lymphocytic infiltration and sensitivity to PD-1 blockade, as demonstrated in KEYNOTE-177 and CheckMate 8HW (30, 31). In contrast, MSS colorectal cancers, which comprise the majority of metastatic CRC, display lower neoantigen loads and limited T-cell infiltration, with objective response rates below 5% to ICI monotherapy (32, 33). Similar immune-resistant phenotypes characterize pancreatic ductal adenocarcinoma and many biliary tract cancers (34, 35).

The tumor microenvironment (TME) represents a central barrier to immune activation across GI tumor types. Multiple interconnected mechanisms contribute to this immunosuppressive state.

Stromal exclusion is particularly pronounced in PDAC, where desmoplastic stroma may account for up to 85% of tumor volume, limiting both therapeutic delivery and immune-cell trafficking (36). Cancer-associated fibroblasts reinforce this barrier through extracellular matrix deposition and secretion of TGF-β, IL-6, and CXCL12. Feig et al. demonstrated that FAP-positive fibroblasts promote T-cell exclusion through CXCL12 signaling, while CXCR4 inhibition restored intratumoral T-cell infiltration and enhanced PD-L1 blockade activity (37).

Immunosuppressive myeloid populations further impair antitumor immunity. In colorectal cancer, tumor-derived CXCL8 promotes M2 macrophage polarization through STAT3 signaling (38). Myeloid-derived suppressor cells inhibit T-cell function through arginase activity and reactive oxygen species production (39), while regulatory T cells suppress immune activation through IL-10, TGF-β, and CTLA-4-mediated signaling.

T-cell exhaustion develops through chronic antigen exposure, leading to progressive upregulation of inhibitory receptors including PD-1, TIM-3, and LAG-3. This phenomenon is well documented in hepatocellular carcinoma, where the tolerogenic hepatic microenvironment, shaped by Kupffer cells, regulatory T cells, and exhausted CD8+ T cells, further limits effective antitumor immunity (40, 41). Similar exhaustion-associated phenotypes have also been observed in MSS colorectal and gastric cancers (42, 43).

Abnormal tumor vasculature provides an additional layer of immune resistance. VEGF signaling restricts lymphocyte trafficking, impairs dendritic-cell maturation, and suppresses cytotoxic T-cell activity (44, 45). Motz et al. demonstrated that tumor-derived VEGF-A induces FasL expression on tumor endothelial cells, selectively eliminating effector CD8+ T cells while sparing regulatory T cells (46).

### 3.2 Resistance to current immunotherapy

Beyond the microenvironmental barriers described above, several tumor-intrinsic mechanisms contribute to immunotherapy resistance in GI cancers.

Oncogenic pathway activation drives immune exclusion. WNT/β-catenin signaling is associated with non-T-cell-inflamed tumor phenotypes and reduced dendritic-cell recruitment (47, 48). KRAS mutations in pancreatic cancer promote immunosuppressive cytokine production and regulatory T-cell conversion, although emerging evidence suggests that KRAS inhibition may partially reverse this suppressive state (49, 50).

Defects in antigen presentation represent another critical resistance mechanism. Alterations involving B2M, JAK1, JAK2, and interferon-γ signaling pathways impair MHC class I expression and reduce tumor recognition by cytotoxic T cells. Zaretsky et al. demonstrated that acquired resistance to PD-1 blockade can emerge through loss-of-function mutations affecting these pathways (51–53). Farah et al. proposed that immunotherapy failure in MSS CRC reflects a “hierarchical immune gating” process in which impaired priming, defective trafficking, and stromal suppression collectively restrict antitumor immunity (54).

Adaptive resistance through compensatory immunosuppressive pathways further compounds these barriers. In colorectal cancer, suppressive myeloid populations including SPP1-high macrophages contribute to T-cell dysfunction (55). In pancreatic cancer, cytokine networks involving IL-6, TGF-β, and macrophage migration inhibitory factors establish self-reinforcing immunosuppressive circuits (56).

Collectively, these mechanisms, including limited tumor immunogenicity, stromal exclusion, oncogenic immune evasion, defective antigen presentation, T-cell exhaustion, and compensatory adaptive resistance, create multiple barriers to effective antitumor immunity in GI cancers. These overlapping resistance pathways help explain why single-pathway immunotherapeutic approaches often fail to produce durable responses. Bispecific antibodies offer a potential solution by simultaneously targeting complementary mechanisms, enabling immune-cell redirection, dual-pathway blockade, localized co-stimulation, and enhanced tumor specificity within a single therapeutic platform.

## 4 Bispecific antibodies in colorectal cancer

### 4.1 Current role of immunotherapy in colorectal cancer

Colorectal cancer (CRC) remains a leading cause of cancer-related mortality worldwide. The clinical benefit of immune checkpoint inhibitors is largely confined to tumors with mismatch repair deficiency (dMMR) or high microsatellite instability (MSI-H), which account for only approximately 4-7% of metastatic CRC cases (29, 30). In the KEYNOTE-177 trial, pembrolizumab significantly improved progression-free survival compared with chemotherapy in MSI-H metastatic CRC (30), while the CheckMate 8HW trial demonstrated superior outcomes with nivolumab plus ipilimumab in this population (29). In contrast, microsatellite-stable (MSS) and proficient mismatch repair (pMMR) tumors, which comprise the vast majority of metastatic CRC, remain largely refractory to checkpoint inhibition, with objective response rates generally below 5% (57, 58).

Liver metastases, present in nearly 70% of patients with metastatic disease, further contribute to treatment resistance by promoting systemic immune tolerance through macrophage-mediated deletion of activated CD8+ T cells (59, 60). Patients without liver metastases appear to derive greater benefit from immunotherapy-based approaches across both MSS and dMMR populations (61). These limitations have fueled interest in bispecific antibodies capable of overcoming immune resistance in CRC, particularly in MSS disease. Carcinoembryonic antigen (CEA), guanylyl cyclase C (GUCY2C), LY6G6D, B7-H3, and immune-modulatory pathways involving PD-1/CTLA-4 and PD-1/VEGF signaling are among the principal targets under investigation (62–65).

### 4.2 CEA-targeted bispecific antibodies

Carcinoembryonic antigen (CEA/CEACAM5) is the most extensively investigated target for bispecific antibody development in colorectal cancer. CEA is overexpressed in more than 98% of colorectal tumors and is largely retained across both MSI-H and MSS disease, making it an attractive target for immune redirection strategies in populations with limited responsiveness to conventional immunotherapy (66, 67).

Most CEA-directed bispecific antibodies function as T-cell engagers, simultaneously binding CEA on tumor cells and CD3ϵ on T lymphocytes to promote antigen-specific cytotoxicity (62, 68). Preclinical studies demonstrated effective T-cell recruitment, enhanced intratumoral immune infiltration, and adaptive PD-L1 upregulation following treatment, providing a rationale for combination approaches with checkpoint inhibitors (62).

Cibisatamab (RG7802/RO6958688) is the most clinically advanced CEA×CD3 bispecific antibody in colorectal cancer. Early-phase studies demonstrated antitumor activity in heavily pretreated CEA-positive solid tumors, including MSS colorectal cancer (69). Clinical activity was enhanced when combined with atezolizumab, supporting the concept that T-cell redirection and checkpoint blockade may act synergistically. Anti-drug antibody formation emerged as a notable limitation, although obinutuzumab pretreatment substantially reduced persistent antibody development (69).

More recently, cibisatamab has been evaluated in combination with FAP-4-1BBL, a fibroblast activation protein-targeted co-stimulatory bispecific antibody designed to enhance T-cell activation within the tumor microenvironment. In patients with MSS metastatic CRC, this approach produced confirmed partial responses, increased intratumoral CD8+ T-cell infiltration, and manageable cytokine release syndrome with optimized dosing schedules (70).

Earlier CEA-targeted BiTE platforms such as MEDI-565 (AMG 211) established proof-of-concept for redirected T-cell therapy in gastrointestinal malignancies but demonstrated limited clinical activity and were complicated by cytokine release syndrome and anti-drug antibody formation (71). Subsequent engineering efforts have focused on optimizing antibody geometry, valency, and CD3 affinity to improve efficacy while minimizing systemic toxicity. Notably, 2 + 1 formats incorporating bivalent CEA binding with monovalent CD3 engagement demonstrated superior tumor-cell killing and T-cell infiltration in preclinical models (63).

Despite continued challenges related to antigen heterogeneity, soluble CEA interference, T-cell exhaustion, stromal exclusion, and on-target gastrointestinal toxicity, CEA-directed bispecific antibodies remain the most clinically advanced bispecific platform in colorectal cancer. Their activity in MSS disease provides important clinical proof-of-concept that T-cell redirection can overcome resistance mechanisms that limit the efficacy of conventional immunotherapy (69, 70, 72).

### 4.3 HER2-directed bispecific approaches

HER2 (ERBB2) amplification or overexpression occurs in approximately 3–5% of metastatic colorectal cancers and is enriched in RAS/BRAF wild-type, left-sided tumors, where prevalence may reach 6-10% (73–75). In addition to functioning as an oncogenic driver, HER2 amplification is a recognized mechanism of resistance to anti-EGFR therapy. A meta-analysis demonstrated significantly inferior outcomes with anti-EGFR treatment in HER2-positive RAS wild-type mCRC, while acquired HER2 amplification has been detected in circulating tumor DNA following cetuximab resistance (76–78).

Clinical interest in HER2-targeted therapy has expanded following the success of dual HER2 blockade and HER2-directed antibody-drug conjugates. Trastuzumab-based combinations demonstrated meaningful activity in the HERACLES-A and MyPathway studies, while tucatinib plus trastuzumab was subsequently established as an effective chemotherapy-free option in refractory HER2-positive mCRC in the MOUNTAINEER trial (74, 79, 80). Trastuzumab deruxtecan also demonstrated substantial activity in DESTINY-CRC02 and is now incorporated into NCCN recommendations for HER2-positive metastatic CRC (73, 81).

Among HER2-directed bispecific antibodies, zanidatamab is the most clinically advanced. Unlike conventional HER2 antibodies that bind a single epitope, zanidatamab simultaneously targets two distinct extracellular HER2 domains corresponding to the trastuzumab and pertuzumab binding sites (82). This biparatopic design enhances receptor clustering, internalization, and degradation while more effectively inhibiting HER2 signaling and promoting immune-mediated cytotoxicity (82, 83). Early clinical studies demonstrated antitumor activity across multiple HER2-positive solid tumors, including colorectal cancer, with manageable toxicity characterized primarily by diarrhea and infusion-related reactions (82). Emerging data suggest that MET amplification may represent an important mechanism of resistance to zanidatamab-based therapy (84).

KN026 is another biparatopic HER2 bispecific antibody targeting the same extracellular HER2 domains (85). Combination strategies integrating KN026 with immunotherapy have shown encouraging activity. In a phase II study, KN026 combined with KN046, a PD-L1/CTLA-4 bispecific antibody, produced promising response rates and durable disease control in heavily pretreated HER2-positive colorectal cancer (86, 87). Biomarker analyses further suggested that CDK12 co-amplification may correlate with improved response, although prospective validation is required (85).

HER2×CD3 T-cell-engaging bispecific antibodies represent an additional area of active investigation. These constructs redirect cytotoxic T cells toward HER2-positive tumor cells independent of endogenous antigen presentation and have demonstrated potent antitumor activity in preclinical colorectal cancer models (88). Newer platforms aim to improve tumor selectivity and reduce toxicity. For example, the nanobody-based HER2 T-cell engager TPP-45142 was engineered to preferentially target HER2-low tumor cells while minimizing toxicity to normal HER2-expressing tissues, including cardiomyocytes (89). Trispecific constructs targeting HER2, HER3, and CD3 are also being explored to address signaling redundancy and enhance immune-cell recruitment (90).

Despite these advances, resistance remains a major challenge. Acquired alterations involving RAS, BRAF, and MET can reactivate downstream MAPK signaling, while HER2 heterogeneity, dynamic loss of amplification, and variable HER2 copy number contribute to treatment failure (75, 84, 91, 92). Nevertheless, HER2-directed bispecific antibodies, particularly biparatopic platforms such as zanidatamab and KN026, represent an important expansion of precision immunotherapy in colorectal cancer and may overcome several limitations associated with conventional HER2-targeted therapies (75, 82, 86).

### 4.4 Other emerging targets in colorectal cancer

Beyond CEA and HER2, several additional targets are being explored for bispecific antibody development in colorectal cancer, particularly in microsatellite-stable (MSS) disease where effective immunotherapeutic options remain limited.

LY6G6D has emerged as a promising target because of its selective overexpression in MSS and MSI-low colorectal cancers and minimal expression in normal tissues (93). LY6G6D×CD3 bispecific antibodies demonstrated potent T-cell activation and antitumor activity in preclinical models, including the ability to induce bystander killing of adjacent antigen-negative tumor cells, potentially mitigating the impact of antigen heterogeneity (94, 95).

GUCY2C is expressed in most primary and metastatic colorectal cancers while remaining largely confined to the apical surface of normal intestinal epithelium, providing a favorable therapeutic window (96, 97). The GUCY2C×CD3 bispecific antibody PF-07062119 demonstrated effective T-cell-mediated killing across multiple colorectal cancer models, including KRAS- and BRAF-mutant tumors, with enhanced activity in combination with checkpoint inhibition and anti-VEGF therapy (65).

B7-H3 represents another attractive target because of its association with immune suppression, angiogenesis, tumor invasion, and poor prognosis (98). Optimized B7-H3×CD3 bispecific antibodies have shown potent antitumor activity in gastrointestinal cancer models while reducing cytokine release through affinity engineering. Additional strategies include B7-H3×4-1BB and EGFR/B7-H3 bispecific constructs designed to augment immune activation while limiting toxicity (99).

EpCAM was among the earliest epithelial targets investigated for T-cell redirection in colorectal cancer. Although initial EpCAM×CD3 constructs demonstrated effective elimination of tumor-initiating cells, clinical development was limited by cytokine release syndrome and on-target toxicity. Current efforts focus on conditionally activated and trispecific platforms designed to improve tumor selectivity and safety (100–102).

Beyond tumor-associated antigens, bispecific strategies are increasingly targeting resistance pathways. EGFR/cMET bispecific antibodies aim to overcome MET-driven resistance to anti-EGFR therapy. Amivantamab demonstrated encouraging activity in heavily pretreated RAS/BRAF wild-type metastatic CRC in the OrigAMI-1 study, including patients with prior anti-EGFR exposure (103, 104). In parallel, dual immune-checkpoint bispecific antibodies, including PD-1/CTLA-4 and PD-1/VEGF constructs, are being evaluated to simultaneously address immune suppression and angiogenesis. Cadonilimab has demonstrated promising activity across advanced solid tumors, while ivonescimab is under investigation in MSS colorectal cancer populations resistant to conventional checkpoint blockade (64, 105).

Together, these emerging platforms illustrate the expanding scope of bispecific antibody development in colorectal cancer. Increasingly, next-generation constructs are designed not only to redirect immune cells but also to overcome antigen heterogeneity, stromal barriers, angiogenesis, and adaptive resistance mechanisms that characterize MSS disease.

### 4.5 Current clinical evidence and ongoing trials

Clinical development of bispecific antibodies in colorectal cancer has accelerated considerably, particularly in microsatellite-stable (MSS) metastatic disease where conventional checkpoint inhibitors have demonstrated limited efficacy. Current approaches extend beyond simple T-cell redirection and increasingly incorporate immune co-stimulation, checkpoint modulation, and targeting of resistance pathways.

CEA-directed bispecific antibodies remain the most advanced class in colorectal cancer. Cibisatamab demonstrated modest single-agent activity but improved efficacy when combined with atezolizumab, supporting synergy between T-cell engagement and checkpoint inhibition (69). More encouraging results were observed with cibisatamab plus FAP-4-1BBL, which enhanced intratumoral T-cell activation and CD8+ T-cell infiltration in heavily pretreated MSS metastatic CRC (70). In parallel, newer T-cell engagers such as the LY6G6D×CD3 bispecific antibody CBI-1214 have entered first-in-human evaluation in advanced MSS/MSI-L CRC (106).

HER2-directed bispecific antibodies have shown promising activity in biomarker-selected populations. Zanidatamab demonstrated clinically meaningful responses in HER2-positive colorectal cancer, including patients previously exposed to HER2-targeted therapies (82). Similarly, the combination of KN026 and KN046 produced encouraging response rates and durable disease control in heavily pretreated HER2-positive disease (86).

Targeting resistance pathways has emerged as another important strategy. Amivantamab demonstrated activity in chemorefractory RAS/BRAF wild-type metastatic CRC, including anti-EGFR-resistant disease, leading to multiple ongoing phase III studies evaluating amivantamab-based combinations (103, 107–109). Likewise, INCA33890, a PD-1×TGFβR2 bispecific antibody, demonstrated encouraging activity in refractory MSS CRC, including patients with liver metastases (110).

Checkpoint/angiogenesis bispecific antibodies are rapidly advancing into late-stage development. Ivonescimab combined with mFOLFOX6 demonstrated promising first-line activity, resulting in initiation of the phase III HARMONi-GI3 trial, while PF-08634404 and pumitamig are also undergoing evaluation in phase III programs (111–114).

Additional platforms seek to enhance immune activation through co-stimulatory signaling. DSP107 combined with atezolizumab demonstrated encouraging disease control in MSS CRC, including patients with liver metastases (115). Multi-bispecific approaches have also entered clinical testing, including combinations of EpCAM×4-1BB and PD-L1×VEGF-A bispecific antibodies administered alongside chemotherapy (116).

Collectively, these studies illustrate the rapid evolution of bispecific antibodies from early proof-of-concept programs toward late-phase clinical development in colorectal cancer. The current pipeline spans tumor-targeting, checkpoint-modulating, angiogenesis-directed, and co-stimulatory platforms, reflecting a shift toward multifunctional immunotherapeutic strategies designed to address the complex biology of MSS colorectal cancer.

## 5 Bispecific antibodies in gastric and gastroesophageal cancers

### 5.1 Current role of immunotherapy in gastric and gastroesophageal cancers

Gastric cancer and gastroesophageal junction (GEJ) adenocarcinoma remain major causes of cancer-related mortality worldwide (117). The addition of PD-1 blockade to platinum- and fluoropyrimidine-based chemotherapy has become a standard first-line approach for many patients with unresectable or metastatic disease, based on the phase III CheckMate-649, KEYNOTE-859, and additional trials evaluating tislelizumab, sintilimab, and sugemalimab (5, 118–120). In HER2-positive disease (~15–25% of gastric cancers), the KEYNOTE-811 trial demonstrated that adding pembrolizumab to trastuzumab and chemotherapy significantly improved outcomes in PD-L1 CPS ≥1 tumors (121, 122). MSI-H/dMMR tumors demonstrate the greatest sensitivity to checkpoint inhibition, while EBV-positive gastric cancers also appear particularly responsive (123, 124).

Despite these advances, most patients eventually develop progressive disease, and durable responses remain limited outside highly selected biomarker-defined populations. The immunosuppressive tumor microenvironment, dynamic changes in PD-L1 and HER2 expression, and adaptive resistance mechanisms provide a strong rationale for bispecific antibody development. In HER2-positive disease, bispecific constructs may improve receptor clustering and maintain activity despite heterogeneous antigen expression (125). The clinical validation of CLDN18.2 through the SPOTLIGHT and GLOW trials has further expanded the landscape of biomarker-directed therapy, with CLDN18.2-directed bispecific antibodies aiming to redirect T-cell cytotoxicity independently of conventional antigen presentation (126, 127).

### 5.2 HER2-directed bispecific antibodies in gastric and gastroesophageal cancers

HER2 overexpression or amplification occurs in approximately 15–20% of gastric and gastroesophageal junction (GEJ) adenocarcinomas and remains one of the most clinically relevant therapeutic targets in this disease (120). Although trastuzumab-based therapy improved outcomes following the ToGA trial, resistance frequently develops, and many tumors exhibit marked spatial and temporal HER2 heterogeneity. Unlike breast cancer, dual HER2 blockade with trastuzumab and pertuzumab failed to significantly improve survival in gastric cancer, highlighting important biologic differences between these malignancies (83). Additional resistance mechanisms include dynamic loss of HER2 expression, epithelial-mesenchymal transition, compensatory HER-family signaling, and activation of downstream PI3K/AKT and MAPK pathways (128–130). These limitations have driven the development of HER2-directed bispecific antibodies designed to achieve more complete receptor inhibition while enhancing antitumor immune activity.

Zanidatamab is currently the most advanced HER2-directed bispecific antibody in gastrointestinal oncology. This biparatopic IgG1 antibody simultaneously binds two distinct extracellular HER2 domains, resulting in enhanced receptor clustering, internalization, and degradation compared with conventional HER2 antibodies (131). In addition to inhibiting HER2 signaling, zanidatamab promotes antibody-dependent cellular cytotoxicity, antibody-dependent cellular phagocytosis, and complement-dependent cytotoxicity, mechanisms that may help overcome resistance associated with incomplete receptor blockade (131).

Early clinical studies demonstrated encouraging activity for zanidatamab in heavily pretreated HER2-expressing gastroesophageal adenocarcinoma, both as monotherapy and in combination with chemotherapy (132). A subsequent first-line phase 2 study combining zanidatamab with chemotherapy reported high response rates and prolonged survival outcomes, with median overall survival exceeding 30 months at long-term follow-up (83). More recently, the phase III HERIZON-GEA-01 trial demonstrated significantly improved progression-free survival with zanidatamab-containing regimens compared with trastuzumab plus chemotherapy in previously untreated HER2-positive metastatic gastroesophageal adenocarcinoma (133). The addition of tislelizumab to zanidatamab and chemotherapy further improved overall survival, supporting the growing rationale for combining HER2-targeted bispecific antibodies with immune checkpoint inhibition.

Additional HER2-directed bispecific platforms are also being explored. KN026, another biparatopic HER2 antibody targeting two distinct HER2 extracellular domains, demonstrated encouraging activity in HER2-positive gastric and GEJ cancers, particularly in tumors with high HER2 expression levels (134). Combination strategies integrating KN026 with KN046, a PD-L1/CTLA-4 bispecific antibody, produced promising response rates in advanced HER2-positive gastrointestinal tumors, suggesting that simultaneous HER2 inhibition and dual-checkpoint modulation may help overcome adaptive immune resistance (87).

Beyond biparatopic HER2 blockade, several emerging platforms aim to directly recruit immune effector cells. HER2×CD3 bispecific antibodies redirect cytotoxic T cells toward HER2-expressing tumor cells independent of conventional antigen presentation pathways. Preclinical studies demonstrated potent activity even in trastuzumab-resistant models and tumors with relatively low HER2 density (135, 136). Similarly, the PD-1/HER2 bispecific antibody IBI315 showed enhanced antitumor activity in preclinical gastric cancer models by simultaneously bridging PD-1-positive T cells with HER2-positive tumor cells and promoting pyroptosis-mediated immune activation (137). Although these T-cell-engaging approaches remain in early-phase development, they represent a promising strategy to overcome immune escape and resistance to conventional HER2-directed therapy.

Despite these advances, important challenges remain. Intratumoral HER2 heterogeneity continues to complicate patient selection and therapeutic durability, while treatment-emergent HER2 loss may reduce the long-term efficacy of HER2-dependent strategies (130). Standardization of HER2 testing in gastric cancer also remains more complex than in breast cancer because of incomplete membranous staining patterns and distinct scoring criteria. In addition, the optimal sequencing of bispecific antibodies with trastuzumab, antibody-drug conjugates, chemotherapy, and checkpoint inhibitors has not yet been established.

Nevertheless, HER2-directed bispecific antibodies represent one of the most clinically advanced and promising areas of bispecific antibody development in gastrointestinal oncology. By combining enhanced receptor targeting with immune activation and improved signaling inhibition, these agents may help overcome several of the biologic limitations that have historically constrained conventional HER2-directed therapy in gastric and gastroesophageal cancers.

### 5.3 CLDN18.2-directed bispecific antibodies in gastric and gastroesophageal cancers

Claudin 18.2 (CLDN18.2) has emerged as one of the most important therapeutic targets in gastric and gastroesophageal junction (GEJ) adenocarcinoma. Under physiologic conditions, CLDN18.2 is confined to differentiated gastric mucosa and remains largely inaccessible within tight junction complexes. During malignant transformation, disruption of epithelial polarity exposes CLDN18.2 on the tumor cell surface, rendering it accessible to antibody-based therapies (127, 138). CLDN18.2 expression has been reported in a substantial proportion of gastric cancers, with approximately 30–50% of tumors meeting the moderate-to-high expression thresholds used in major clinical trials (139). Importantly, CLDN18.2 expression appears largely independent of HER2, PD-L1, and mismatch repair status, suggesting that it defines a distinct and complementary therapeutic subgroup (140, 141).

The therapeutic relevance of CLDN18.2 was clinically validated by the phase III SPOTLIGHT and GLOW trials, which demonstrated that zolbetuximab combined with chemotherapy significantly improved progression-free survival (PFS) and overall survival (OS) in patients with CLDN18.2-positive, HER2-negative advanced gastric or GEJ adenocarcinoma (119, 142). These studies established CLDN18.2 as a clinically actionable biomarker and led to FDA approval of zolbetuximab in 2024. However, zolbetuximab primarily relies on Fc-mediated immune effector mechanisms such as antibody-dependent cellular cytotoxicity (ADCC) and complement-dependent cytotoxicity (CDC), and its activity may therefore be limited by the immunosuppressive tumor microenvironment commonly observed in gastric cancer (143). These limitations have accelerated interest in CLDN18.2-directed bispecific antibodies capable of directly recruiting and activating immune cells within the tumor microenvironment.

Among emerging strategies, CLDN18.2×CD3 T-cell engagers represent the most actively investigated platform. Preclinical studies demonstrated potent T-cell-mediated cytotoxicity against gastric cancer models, including tumors with low antigen density and immunologically “cold” microenvironments (144, 145). AMG 910, a half-life extended CLDN18.2-targeted bispecific T-cell engager, showed strong antitumor activity in gastric cancer xenografts while also promoting regulatory T-cell reprogramming toward a more pro-inflammatory phenotype (144). Similarly, affinity-optimized constructs such as AZD5863 demonstrated effective tumor killing with lower cytokine release, suggesting the potential for improved safety profiles (146).

Encouraging activity has also been reported in early-phase clinical studies. IBI389, an anti-CLDN18.2×CD3 bispecific antibody, demonstrated manageable safety and preliminary efficacy in heavily pretreated gastric and GEJ cancers (126). Cytokine release syndrome (CRS) was common but predominantly low grade, with limited severe toxicity and no treatment-related deaths reported. In CLDN18.2-positive gastric and GEJ tumors, IBI389 achieved meaningful disease control and durable responses despite advanced disease status. Similarly, QLS31905 combined with chemotherapy demonstrated high response rates in first-line CLDN18.2-positive gastric cancer, including activity in tumors with relatively low CLDN18.2 expression, suggesting that T-cell-engaging bispecific antibodies may broaden the population eligible for CLDN18.2-directed therapy beyond the strict thresholds currently required for zolbetuximab (147).

Additional platforms are now integrating CLDN18.2 targeting with checkpoint inhibition or co-stimulatory signaling. Q-1802, a bispecific antibody targeting CLDN18.2 and PD-L1, was designed to combine direct tumor targeting with local immune checkpoint blockade (148). Early clinical data demonstrated high response rates and prolonged disease control when combined with chemotherapy in treatment-naive CLDN18.2-positive gastric and GEJ cancers, particularly in tumors with high CLDN18.2 expression and elevated PD-L1 CPS. Importantly, toxicity appeared manageable, with relatively low discontinuation rates.

A mechanistically distinct strategy is represented by givastomig, a CLDN18.2×4-1BB bispecific antibody that provides tumor-localized co-stimulation rather than direct CD3-mediated T-cell engagement. By activating 4-1BB signaling only in the presence of CLDN18.2-expressing tumor cells, givastomig aims to enhance antitumor immunity while minimizing systemic immune activation (149). Early-phase studies demonstrated acceptable tolerability and preliminary antitumor activity in CLDN18.2-positive gastroesophageal cancers, supporting ongoing development in combination with checkpoint inhibitors and chemotherapy. Similar approaches are being explored with additional CLDN18.2×4-1BB constructs such as PM1032 (150).

Despite these advances, several important challenges remain. CLDN18.2 expression demonstrates substantial intratumoral heterogeneity, with discordant expression frequently observed between primary tumors and metastatic sites (151, 152). Chemotherapy-induced loss of CLDN18.2 expression has also been reported, raising concerns regarding treatment-emergent target escape (151). In addition, variability in immunohistochemical assays, staining platforms, and positivity thresholds complicates cross-trial comparisons and patient selection (67). Current studies employ markedly different expression cutoffs, ranging from low-level positivity to the high-expression criteria used in zolbetuximab trials, highlighting the need for standardized biomarker assessment.

Toxicity profiles also differ significantly across CLDN18.2-directed platforms. Fc-mediated antibodies such as zolbetuximab are primarily associated with gastrointestinal adverse events including nausea and vomiting, whereas CD3-engaging bispecific antibodies more commonly produce CRS, lymphocytopenia, and immune-mediated toxicities. Optimization of dosing strategies, affinity engineering, and step-up dosing protocols will therefore remain critical for improving the therapeutic index of these agents.

Overall, CLDN18.2-directed bispecific antibodies represent one of the most promising emerging therapeutic strategies in gastric and GEJ cancers. By combining tumor targeting with direct immune-cell recruitment, checkpoint modulation, or conditional co-stimulation, these platforms may overcome several limitations of conventional monoclonal antibodies and expand the benefits of targeted immunotherapy to a broader population of patients with immunologically resistant disease. Ongoing phase III studies and expanding early-phase programs will further define the role of CLDN18.2-directed bispecific antibodies within the evolving treatment landscape of gastrointestinal oncology.

### 5.4 Other emerging bispecific antibody targets in gastric and gastroesophageal cancers

Although HER2- and CLDN18.2-directed bispecific antibodies are currently the most developed platforms in gastric and gastroesophageal junction (GEJ) cancers, several additional strategies are emerging. These agents aim to overcome immune exclusion, angiogenesis-driven suppression, adaptive checkpoint resistance, and limited T-cell activation by integrating checkpoint blockade, vascular modulation, co-stimulation, or innate immune activation within single therapeutic constructs.

B7-H3 has gained attention as an immunoregulatory target in gastric cancer because it is expressed on tumor cells and cancer-associated fibroblasts, has limited expression in normal tissues, and is associated with advanced disease and poor survival (153). Mechanistically, B7-H3 contributes to immune evasion, angiogenesis, epithelial-mesenchymal transition, invasion, and chemoresistance (154, 155). These features have supported the development of B7-H3-directed bispecific antibodies. Optimized B7-H3×CD3 constructs such as CC-3 demonstrated potent T-cell activation and tumor-cell killing with reduced cytokine release through CD3 affinity tuning (99). Similar activity was later shown across gastric, pancreatic, and hepatic cancer models (156). Other platforms, including EX105 and Fc-engineered NKG2D×B7-H3 bispecific antibodies, are being developed to improve immune-cell recruitment while maintaining a favorable safety profile (157, 158).

Angiogenesis-integrated bispecific antibodies represent another important direction. VEGF signaling contributes not only to tumor vascularization but also to immune suppression through impaired dendritic-cell maturation, reduced cytotoxic T-cell infiltration, and expansion of regulatory T cells and myeloid-derived suppressor cells (159, 160). Ivonescimab, a PD-1×VEGF bispecific antibody, was designed to combine checkpoint blockade with anti-angiogenic activity in a single construct. Early clinical studies demonstrated sustained PD-1 receptor occupancy, VEGF suppression, and manageable toxicity across advanced solid tumors, and ongoing trials are evaluating ivonescimab-based combinations in advanced gastroesophageal adenocarcinoma (159, 161, 162). The combination of ivonescimab with cadonilimab and chemotherapy has also shown encouraging early activity in untreated gastric and GEJ adenocarcinoma, including patients with liver or peritoneal metastases (163). Pumitamig, a PD-L1×VEGF-A bispecific antibody, is similarly being evaluated with chemotherapy and antibody-drug conjugates in gastrointestinal malignancies.

Dual checkpoint bispecific antibodies are also gaining clinical relevance. Cadonilimab, a PD-1×CTLA-4 bispecific antibody, is the most advanced agent in this category. By localizing CTLA-4 blockade preferentially to PD-1-positive activated T cells, cadonilimab may provide broader immune activation with a more manageable safety profile than conventional dual-checkpoint combinations (64). In the phase III COMPASSION-15 trial, cadonilimab plus chemotherapy improved overall survival, progression-free survival, and objective response rate compared with chemotherapy alone in advanced HER2-negative gastric or GEJ adenocarcinoma, with benefit observed regardless of PD-L1 expression (164). Cadonilimab-based regimens are also being explored in neoadjuvant settings and in combination with macrophage checkpoint blockade (165, 166).

Additional dual-checkpoint platforms are expanding beyond the PD-1/CTLA-4 axis. Volrustomig, a PD-1/CTLA-4 bispecific antibody with preferential CTLA-4 targeting on PD-1-positive T cells, has shown early antitumor activity across solid tumors and is being evaluated in gastric cancer platform trials (167, 168). Rilvegostomig, a PD-1/TIGIT bispecific antibody, and AZD7789, a PD-1/TIM-3 bispecific antibody, are also under investigation in advanced gastric and GEJ cancers, reflecting growing interest in targeting compensatory exhaustion pathways.

Innate immune checkpoint and co-stimulatory platforms represent another developing area. The CD47-SIRPα axis enables tumor cells to evade macrophage-mediated phagocytosis, and CD47 expression has been associated with unfavorable outcomes in gastric cancer (169, 170). Preclinical co-targeting of CD47 and VEGF demonstrated strong antitumor effects in gastric cancer models (170). Clinically, combinations of cadonilimab with the anti-CD47 antibody AK117 and chemotherapy have shown promising early activity in advanced gastric and GEJ cancer (1^165^ ASCO). Other agents, such as HCB101, are being explored with ramucirumab and paclitaxel in later-line disease (171, 172).

Finally, tumor-localized co-stimulatory bispecific antibodies are being designed to enhance immune activation while avoiding systemic toxicity. 4-1BB-based constructs, including CLDN18.2×4-1BB and EpCAM×4-1BB platforms, aim to restrict co-stimulation to antigen-positive tumor sites and may complement checkpoint inhibition or chemotherapy (116, 149). Overall, these emerging platforms reflect a shift from single-pathway immunotherapy toward multifunctional designs that combine tumor targeting, checkpoint blockade, angiogenesis inhibition, innate immune activation, and co-stimulation. Most remain in early clinical development, and their future role will depend on biomarker refinement, toxicity management, and rational integration with chemotherapy, antibody-drug conjugates, and established checkpoint inhibitors.

Clinical development is increasingly shifting toward platform trial designs, exemplified by the GEMINI-Gastric trial (NCT05702229), which simultaneously investigates multiple bispecific and immune-modulating combinations for first-line HER2-negative gastric cancer, and the phase III ARTEMIDE-Gastric01 trial, which combines rilvegostomig with trastuzumab deruxtecan in HER2-positive disease (167, 173).

### 5.5 Safety and toxicity considerations of bispecific antibodies

The expanding diversity of bispecific antibody (BsAb) platforms in gastrointestinal (GI) oncology has created a heterogeneous toxicity landscape. Unlike conventional monoclonal antibodies, BsAbs may simultaneously engage immune effector cells, activate co-stimulatory pathways, inhibit multiple checkpoints, or target more than one tumor antigen. Consequently, adverse events vary substantially according to antibody format and mechanism of action. In addition, many GI-associated targets, including CEA, CLDN18.2, GUCY2C, and HER2, are expressed to varying degrees on normal tissues, creating an inherent risk of on-target/off-tumor toxicity (9). A recent meta-analysis of T-cell engagers (TCEs) in solid tumors identified gastrointestinal and inflammatory toxicities as the most common adverse events and suggested that Fc-independent constructs may be associated with lower inflammatory toxicity (174).

#### 5.5.1 Cytokine release syndrome and neurotoxicity

Cytokine release syndrome (CRS) remains the hallmark toxicity of CD3-engaging BsAbs and results from rapid T-cell activation with subsequent release of IL-6, IFN-γ, TNF-α, and other inflammatory mediators (175). CRS typically occurs within the first few days after treatment initiation and is most frequently observed during early step-up dosing cycles (176).

Among GI-directed TCEs, cibisatamab (CEA×CD3) combined with FAP-4-1BBL induced CRS in 57.7% of patients, although grade ≥3 CRS occurred in only 3.8%; implementation of a lower cycle 1 dose eliminated grade ≥3 CRS events (70). Similarly, IBI389 (CLDN18.2×CD3) was associated with CRS in 59.5% of treated patients, but only one grade 3 event was reported (126). In contrast, co-stimulatory 4-1BB-based bispecific antibodies such as BGB-B167 have shown no CRS signal to date, highlighting a major safety distinction between TCEs and non-CD3 platforms (177).

Immune effector cell-associated neurotoxicity syndrome (ICANS) appears uncommon in GI-directed BsAbs. No neurotoxicity was reported in studies of cibisatamab, IBI389, or QLS31905, and real-world analyses have shown grade ≥3 ICANS rates of only 0.4-1.3% (69, 126, 178). Nevertheless, continued monitoring remains important as larger studies mature.

A multicenter real-world analysis of 3,642 patients receiving BsAbs across tumor types reported severe CRS rates of only 0.5%, 0.8%, and 1.0% at 7, 30, and 90 days, respectively, with tocilizumab use ranging from 6.7% to 9.8% (178). However, both clinical and real-world studies likely underestimate CRS incidence because grading systems vary and mild CRS can be difficult to distinguish from infusion-related reactions (174).

#### 5.5.2 On-target/off-tumor toxicity

On-target/off-tumor toxicity remains one of the greatest barriers to successful BsAb development in GI cancers because many target antigens are also expressed on normal gastrointestinal tissues. Cibisatamab caused colitis in 13.5% of treated patients, including one fatal case of cytomegalovirus-associated colitis (70). Complementary organoid studies demonstrated dose-dependent killing of healthy CEA-expressing tissues, confirming the biological basis of this toxicity (179).

For CLDN18.2-targeted therapies, gastric toxicity represents the principal concern. Clinical experience with zolbetuximab has demonstrated substantial nausea and vomiting attributable to normal gastric mucosal expression of CLDN18.2, while preclinical studies have shown that lower-affinity binders can reduce off-tumor gastric injury and improve the therapeutic window (142, 180). Similarly, GUCY2C-targeted cellular therapies have been associated with significant diarrhea, reflecting expression of GUCY2C on normal intestinal epithelium (181).

Hematologic toxicities are also observed across several platforms. IBI389 produced grade ≥3 lymphocytopenia in 14.0% of patients (126), whereas the CLDN18.2/PD-L1 bispecific Q-1802 was associated with grade ≥3 thrombocytopenia (8.1%), neutropenia (6.5%), and anemia (4.8%) (148). Cadonilimab-based regimens have similarly been associated with substantial rates of neutropenia and leukopenia when combined with chemotherapy (182).

#### 5.5.3 Hepatotoxicity and gastrointestinal adverse events

Hepatotoxicity remains an important concern for certain BsAb platforms. The most striking example comes from intravenous catumaxomab, which caused fatal acute liver failure through Fc-mediated activation of Kupffer cells and secondary T-cell recruitment, despite the absence of EpCAM expression on hepatocytes (183). This observation has strongly influenced subsequent Fc-engineering strategies. More recently, IBI389 was associated with grade ≥3 gamma-glutamyl transferase elevation in 21.5% of patients (126), while the GPC3×4-1BB bispecific BGB-B2033 demonstrated occasional grade ≥3 ALT, AST, and bilirubin elevations (184). In contrast, tumor-conditional 4-1BB agonists such as givastomig and BGB-B167 have shown limited hepatotoxicity, suggesting that localized co-stimulatory activation may improve safety (149, 177).

Gastrointestinal toxicity is the most prevalent adverse-event category across GI-directed BsAbs. Diarrhea represents the dominant toxicity of zanidatamab, occurring in up to 52% of patients in early studies and accounting for the majority of grade ≥3 treatment-related adverse events in later trials (82, 83, 133). Importantly, implementation of mandatory antidiarrheal prophylaxis reduced grade 3 diarrhea rates from 39% to 24%, illustrating how toxicity-management strategies can evolve during clinical development (83).

#### 5.5.4 Anti-drug antibodies

Immunogenicity has emerged as an additional challenge for some TCEs. In phase I studies of cibisatamab, persistent anti-drug antibodies developed in 40–52% of patients and were associated with reduced drug exposure. Pretreatment with obinutuzumab reduced persistent ADA formation to 8%, highlighting a potential strategy for mitigating immunogenicity (69).

#### 5.5.5 Platform-specific toxicity profiles

These differences highlight the importance of considering BsAbs as distinct therapeutic classes rather than a single drug category. T-cell engagers are primarily limited by CRS and on-target/off-tumor toxicity, whereas checkpoint- and angiogenesis-based BsAbs generally exhibit toxicity profiles resembling those of their parent monoclonal antibodies. Co-stimulatory BsAbs appear to occupy an intermediate position, with reduced CRS risk but continued concerns regarding immune-mediated and hepatic adverse events.

#### 5.5.6 Engineering strategies to improve safety

Several engineering approaches are being developed to expand the therapeutic window of BsAbs. Step-up dosing has become standard for CD3-engaging constructs and has significantly reduced severe CRS rates in clinical studies (70). Affinity tuning of the CD3-binding arm can decrease cytokine release while maintaining antitumor activity, although the optimal affinity appears target-dependent (99, 187). Additional strategies include Fc engineering to prevent Fcγ receptor-mediated toxicity, protease-activatable masked antibodies that become active only within the tumor microenvironment, and tumor-conditional co-stimulatory designs that restrict immune activation to antigen-positive tissues (188–190).

#### 5.5.7 Controversies and unresolved questions

Despite encouraging advances, several unresolved challenges remain. CRS reporting remains inconsistent across studies because different grading systems are used and mild events may be under-recognized (174, 178). On-target/off-tumor toxicity continues to limit the development of TCEs directed against antigens expressed on normal GI tissues, and the optimal balance between affinity reduction and efficacy has not yet been established clinically (187). In addition, long-term immunologic consequences, including infectious complications and chronic immune dysfunction, remain poorly characterized in GI oncology populations. As bispecific antibodies move into larger randomized trials and earlier lines of therapy, standardized toxicity reporting and systematic long-term safety monitoring will become increasingly important.

## 6 Bispecific antibodies in hepatocellular carcinoma

### 6.1 Current role of immunotherapy in hepatocellular carcinoma

Hepatocellular carcinoma (HCC) is the most common primary liver malignancy and a leading cause of cancer-related mortality worldwide (191). The treatment landscape for advanced HCC has been transformed by the integration of immune checkpoint inhibitors with anti-angiogenic agents and dual checkpoint combinations. The phase III IMbrave150 trial established atezolizumab plus bevacizumab as the first practice-changing immunotherapy regimen (median OS 19.2 vs. 13.4 months with sorafenib; HR 0.66) (192, 193). The HIMALAYA trial subsequently demonstrated improved OS with the STRIDE regimen (tremelimumab plus durvalumab) compared with sorafenib (median OS 16.4 vs. 13.8 months; HR 0.78), with five-year OS rates of 19.6% versus 9.4% (194, 195). More recently, CheckMate 9DW demonstrated improved OS with nivolumab plus ipilimumab versus lenvatinib or sorafenib (median OS 23.7 vs. 20.6 months; HR 0.79; ORR 36% vs. 13%), although higher early mortality and significant immune-mediated toxicity were observed (196).

Current NCCN guidelines list atezolizumab plus bevacizumab, durvalumab plus tremelimumab, and ipilimumab plus nivolumab as preferred first-line systemic therapy options (197). Despite these advances, a substantial proportion of patients either fail to respond or develop progressive disease. The tolerogenic hepatic microenvironment characterized by Kupffer cell-mediated immune suppression, regulatory T-cell enrichment, and exhausted CD8+ T-cell populations contributes to both primary and acquired resistance. No validated predictive biomarkers currently exist to guide immunotherapy selection in HCC, and optimal treatment sequencing after first-line immunotherapy remains undefined (198, 199). These limitations have driven interest in bispecific antibodies, with glypican-3 (GPC3) emerging as the dominant tumor-associated target.

### 6.2 GPC3-directed bispecific antibodies

Glypican-3 (GPC3) is a cell-surface heparan sulfate proteoglycan overexpressed in approximately 70% of HCC cases while remaining largely absent from normal adult liver tissue, making it one of the most attractive tumor-specific targets for immunotherapy in this disease (200, 201). GPC3 promotes tumor growth through activation of Wnt/β-catenin and other oncogenic signaling pathways, and its expression has been associated with poor prognosis (202). Despite its promise as a target, monoclonal antibody approaches directed against GPC3 have yielded disappointing clinical results. The humanized anti-GPC3 antibody GC33 showed no significant improvement in overall survival or progression-free survival compared with placebo in a phase II trial, highlighting the need for more potent therapeutic strategies (203).

GPC3×CD3 bispecific T-cell engagers represent the most extensively investigated bispecific platform in HCC. Multiple preclinical studies have demonstrated potent, GPC3-specific T-cell activation and tumor-cell killing across HCC cell lines and xenograft models (204–206). Importantly, membrane-proximal epitope targeting has emerged as a critical engineering consideration. GPC3 C-terminal glycosylation can mask epitopes and compromise antibody binding, prompting the development of N-terminal-directed constructs that avoid glycosylation interference while simultaneously modulating Wnt/β-catenin and PI3K/AKT signaling (207). Combination of GPC3×CD3 bispecific antibodies with irinotecan dramatically improved efficacy and arrested tumor growth in xenograft models, supporting combination-based strategies (204).

SAR444200 represents a novel NANOBODY-based T-cell engager targeting TCRαβ rather than CD3, which may offer specific advantages including broader T-cell engagement and potentially improved safety. Preclinical evaluation demonstrated picomolar potency against multiple GPC3-positive tumor cell lines and complete tumor regression in xenograft models at doses starting from 0.7 mg/kg, with favorable tolerability in non-human primates up to 8 mg/kg (208). In the first-in-human phase 1/2 study (NCT05450562), SAR444200 was tolerated across multiple dose levels in patients with GPC3-positive advanced solid tumors, predominantly HCC. Stable disease was observed in 30.3% of patients, and among HCC patients with elevated baseline alpha-fetoprotein, 27% showed ≥50% AFP reduction, suggestive of preliminary antitumor activity (209).

AZD9793, a first-in-class CD8-guided trispecific T-cell engager targeting GPC3, has also entered clinical evaluation. This construct preferentially engages CD8+ T cells while minimizing CD4+ T-cell activation and unwanted cytokine release, combining bivalent GPC3 binding with CD8-biased engagement and low-affinity T-cell receptor binding. The RHEA-1 first-in-human phase I/II trial (NCT06795022) is currently enrolling patients with GPC3-positive advanced or metastatic HCC (210).

An innovative organ-specific delivery approach has also been developed. MTS105, an mRNA-encoded GPC3-targeted T-cell engager delivered via lipid nanoparticles, achieves preferential liver expression and higher intratumoral drug exposure compared with conventional antibody-based constructs. In preclinical models, MTS105 induced complete, dose-dependent tumor regression in liver-orthotopic HCC models with rapid intratumoral T-cell activation and favorable pharmacokinetics in cynomolgus monkeys, supporting advancement into a first-in-human study currently underway (211).

BGB-B2033 (GPC3×4-1BB) represents a mechanistically distinct approach. Rather than directly engaging CD3, this bispecific antibody provides tumor-localized co-stimulation by activating 4-1BB signaling only in the presence of GPC3-expressing tumor cells. In a first-in-human phase 1 study enrolling predominantly second-line or later HCC patients, BGB-B2033 demonstrated a confirmed objective response rate of 20.3% (95% CI: 11.0–32.8), with 10 of 12 responders having ongoing responses at data cutoff (184). At doses above the predicted target efficacious dose, the confirmed ORR increased to 28.9%. The agent was generally well tolerated, with grade ≥3 treatment-related adverse events occurring in only 8.2% of patients and immune-mediated adverse events limited to grades 1–2. Triplet dose escalation with tislelizumab and bevacizumab is ongoing (184).

Despite encouraging early clinical activity, several challenges remain. Variability in GPC3 expression across tumors, the absence of standardized companion diagnostic assays, and uncertainty regarding optimal expression thresholds may complicate patient selection and clinical trial design. As GPC3-directed therapies advance through clinical development, biomarker standardization will become increasingly important for identifying patients who are most likely to benefit from these approaches.

### 6.3 Dual checkpoint and anti-angiogenic bispecific strategies in HCC

Beyond GPC3-directed approaches, several bispecific antibodies targeting immune checkpoint and angiogenic pathways are entering clinical development in HCC. These strategies aim to improve upon existing combination regimens by integrating checkpoint blockade and anti-angiogenic activity within a single construct. The biologic rationale for combining PD-1 blockade with VEGF inhibition in HCC is well established: dual PD-1/VEGFR-2 blockade reprograms the immune microenvironment by increasing CD8+ T-cell infiltration, shifting macrophage polarization, reducing regulatory T-cell infiltration, and promoting vascular normalization, resulting in doubled survival in preclinical HCC models (212).

GPC3×PD-L1 bispecific antibodies have been developed to provide tumor-directed checkpoint inhibition. Yuan et al. (213) engineered a construct with high GPC3 affinity but approximately 300-fold reduced PD-L1 affinity compared with atezolizumab, enabling GPC3-dependent PD-1/PD-L1 blockade that selectively targets GPC3/PD-L1 double-positive tumors while sparing PD-L1-positive normal tissues. Biodistribution studies confirmed preferential accumulation in GPC3-positive tumors, providing proof-of-concept for tumor-targeted checkpoint inhibition in HCC (213).

GPC3×CD47 bispecific antibodies represent another promising strategy that engages innate immunity. Du et al. (214) demonstrated that a GPC3/CD47 bispecific antibody induced enhanced Fc-mediated effector functions against dual antigen-expressing HCC cells, with both macrophages and neutrophils required for its strong efficacy. Importantly, this construct outperformed both monotherapies and the combination of anti-CD47 and anti-GPC3 monoclonal antibodies in xenograft models, while maintaining an extended serum half-life without systemic toxicity in humanized mice (214).

GPC3×CD16A bispecific antibodies redirect natural killer cells toward GPC3-positive tumor cells. Chen et al. (215) developed an Fc-stabilized construct that demonstrated potent, dose-dependent NK cell-mediated cytotoxicity against multiple HCC cell lines, surpassing conventional ADCC. Synergistic tumor suppression was observed when combined with sorafenib, suggesting potential integration with existing systemic therapy (215).

PD-1/TIGIT bispecific antibodies are also being evaluated in HCC. Nilvanstomig (ZG005), an anti-PD-1/TIGIT bispecific antibody, was evaluated in combination with bevacizumab versus sintilimab plus bevacizumab biosimilar as first-line therapy for advanced HCC in a randomized phase II trial. With a median follow-up of approximately 5 months, the ZG005-based arms demonstrated higher response rates (ORR 32.3–37.5% vs. 25.0% per RECIST v1.1; 50.0–54.8% vs. 34.4% per mRECIST) and prolonged progression-free survival compared with the control arm (HR 0.28–0.40), with comparable safety profiles (216).

Ivonescimab (PD-1/VEGF bispecific antibody) combined with hepatic arterial infusion chemotherapy (HAIC) has also demonstrated encouraging early activity in first-line unresectable HCC, with an ORR of 41.7% per RECIST v1.1 and a surgical conversion rate of 58.3% (217).

### 6.4 Clinical evidence and ongoing trials

Clinical development of bispecific antibodies in HCC is rapidly expanding across multiple mechanistic platforms. The following table summarizes key agents currently under investigation.

Multiple bispecific antibody platforms are now advancing through clinical development in HCC, reflecting growing efforts to overcome the limitations of conventional immunotherapy. GPC3 remains the dominant tumor-associated target, while additional strategies are expanding to include dual checkpoint inhibition, anti-angiogenic combinations, innate immune checkpoint blockade, NK-cell redirection, and tumor-selective immune activation. Ongoing clinical studies will help define the optimal integration of these agents with established immunotherapy regimens and determine which patient populations are most likely to derive meaningful benefit.

## 7 Bispecific antibodies in pancreatic cancer

### 7.1 Current treatment landscape and immunotherapy resistance in pancreatic cancer

Pancreatic ductal adenocarcinoma (PDAC) remains one of the most lethal malignancies, with a 5-year survival rate of less than 13% (218). Systemic chemotherapy remains the cornerstone of treatment for advanced disease, with FOLFIRINOX and gemcitabine plus nab-paclitaxel as first-line regimens and fluorouracil/leucovorin/liposomal irinotecan as a subsequent-line option (219).

PDAC has demonstrated remarkable resistance to immunotherapy. Clinical trials evaluating checkpoint inhibitors, either as monotherapy or in combination, have generally failed to produce meaningful benefit in unselected populations (220, 221). The role of immunotherapy is currently restricted to the approximately 1–2% of patients harboring MSI-H/dMMR tumors, for whom pembrolizumab, dostarlimab, and nivolumab plus ipilimumab represent guideline-supported options (219). Zenocutuzumab, a HER2×HER3 bispecific antibody functioning as an NRG1 ligand trap, recently received FDA approval for NRG1 fusion-positive PDAC, notably representing a successful bispecific antibody application in this disease, although its mechanism differs from the immune-redirecting and checkpoint-modulating platforms that are the primary focus of this review (222).

The profound immunotherapy resistance of PDAC reflects its uniquely immunosuppressive tumor microenvironment, including dense desmoplastic stroma, cancer-associated fibroblast-mediated immune exclusion, KRAS-driven immunoregulatory signaling, and accumulation of suppressive myeloid cells and regulatory T cells. These barriers have made PDAC a particularly important setting for bispecific antibody development, with mesothelin, CLDN18.2, CEA, B7-H3, and stromal targets under active investigation (223, 224).

### 7.2 Mesothelin-directed bispecific antibodies

Mesothelin (MSLN) is one of the most extensively investigated immunotherapeutic targets in pancreatic ductal adenocarcinoma (PDAC). This glycosylphosphatidylinositol-anchored cell-surface glycoprotein is overexpressed in approximately 80–100% of PDACs while exhibiting only limited expression on normal mesothelial tissues (225–228). In a tissue microarray study of 599 pancreatic cancers, mesothelin expression was detected in 89% of pancreatic adenocarcinomas and was absent in normal pancreatic tissue (228). Its high prevalence and relative tumor specificity have made mesothelin an attractive target for antibody-based therapies, although earlier monoclonal antibody and immunotoxin strategies achieved only limited clinical success (226, 229).

A major obstacle to mesothelin-targeted therapy is antigen shedding. Proteolytic cleavage of mesothelin releases soluble mesothelin (sMSLN) into the tumor microenvironment and circulation, where it can act as an antigen sink that sequesters therapeutic antibodies and reduces tumor targeting (27, 28). In addition, high mesothelin expression has been associated with an immunologically unfavorable microenvironment characterized by reduced cytotoxic T-cell infiltration, suggesting that successful mesothelin-directed therapies may need to overcome both antigen-related and microenvironmental barriers (230).

To address antigen shedding, several next-generation bispecific antibodies have been engineered to recognize membrane-proximal epitopes that remain attached to the tumor cell surface. JNJ-79032421 was specifically designed to bind the membrane-restricted, non-shed C-terminal region of mesothelin. Structural studies confirmed selective recognition of this membrane-proximal epitope, and both binding affinity and cytotoxic activity were preserved in the presence of soluble mesothelin, unlike conventional mesothelin-targeting constructs whose activity was substantially reduced by shed antigen (28). In both cell-line-derived and patient-derived xenograft models, higher levels of mesothelin expression correlated with greater therapeutic potency, accompanied by dose-dependent increases in intratumoral CD45+ and CD8+ immune-cell infiltration and enhanced T-cell activation (28).

Similar findings have been reported with other membrane-proximal targeting approaches. Chakraborty et al. developed a 15B6-derived mesothelin×CD3 bispecific antibody directed against the protease-sensitive C-terminal region of mesothelin, demonstrating sustained antitumor activity despite the presence of recombinant shed mesothelin both *in vitro* and *in vivo* (27). Likewise, Hatterer et al. showed that targeting membrane-proximal mesothelin epitopes enhanced both antibody-dependent cellular cytotoxicity (ADCC) and antibody-dependent cellular phagocytosis (ADCP). A bispecific construct combining membrane-proximal mesothelin recognition with CD47 blockade demonstrated superior antitumor activity compared with membrane-distal targeting strategies in xenograft models (231).

Several mesothelin-directed T-cell engagers have now advanced into clinical development. HPN536 is a 53-kDa trispecific T-cell-activating construct that simultaneously binds mesothelin on tumor cells, CD3ϵ on T cells, and serum albumin to prolong systemic exposure. Preclinical studies demonstrated potent redirected T-cell cytotoxicity and robust mesothelin-dependent activity across multiple tumor models. In cynomolgus monkeys, doses ranging from 0.1 to 10 mg/kg were well tolerated and generated pharmacokinetic profiles supportive of weekly dosing schedules. These findings led to initiation of an ongoing phase I clinical trial (NCT03872206) in patients with mesothelin-expressing malignancies (232).

Additional platforms continue to expand the mesothelin-targeted landscape. NAV-003, a divalent MSLN×CD3ϵ bispecific antibody targeting a membrane-proximal epitope, demonstrated enhanced cytotoxicity against immunosuppressive protein-producing tumor cells and significant activity in patient-derived mesothelioma xenograft models co-engrafted with human peripheral blood mononuclear cells (233). NM28-2746, a bivalent mesothelin-directed T-cell engager, was designed to preferentially target tumors with high mesothelin expression while minimizing activity against low-expressing normal tissues. In pancreatic cancer xenograft models, antitumor activity was further enhanced when NM28-2746 was combined with NM21-1480, a PD-L1/4-1BB bispecific antibody that provides localized co-stimulatory signaling (234). More recently, ZW171, a trivalent mesothelin×CD3 bispecific antibody, entered first-in-human evaluation (NCT06523803) in patients with advanced mesothelin-expressing solid tumors, including PDAC, using a subcutaneous step-up dosing strategy designed to improve tolerability (235).

Beyond conventional CD3-mediated T-cell engagement, alternative immune effector strategies are also being explored. Zhu et al. demonstrated that a mesothelin/CD3 bispecific antibody could effectively activate both natural killer T (NKT) cells and γδ T cells, producing stronger antitumor responses than conventional peripheral blood mononuclear cells while generating relatively low cytokine release in pancreatic cancer models (236). These findings suggest that harnessing non-conventional lymphocyte populations may offer a potential strategy to improve efficacy while reducing some of the toxicity concerns associated with traditional CD3-directed bispecific antibodies.

Collectively, mesothelin-directed bispecific antibodies represent one of the most mature and diverse therapeutic strategies under investigation in PDAC. Advances in epitope selection, particularly the shift toward membrane-proximal targeting, appear capable of overcoming the longstanding challenge of antigen shedding. At the same time, the emergence of trispecific constructs, co-stimulatory combinations, and alternative immune-cell engagement strategies reflects ongoing efforts to address the profound immune suppression that characterizes pancreatic cancer. Whether these innovations can translate into meaningful clinical benefit remains an important question currently being addressed in early-phase clinical trials.

### 7.3 CLDN18.2 and other emerging targets in pancreatic cancer

CLDN18.2 expression has been reported in a substantial proportion of pancreatic ductal adenocarcinomas. A systematic review and meta-analysis of 16 studies including 2,025 PDAC patients found a pooled prevalence of CLDN18.2 expression of 51.60% (95% CI: 40.93–62.19), with substantial heterogeneity across studies (I² = 95.4%) attributable in part to differences in antibody clones used for immunohistochemistry (237). A large real-world cohort study found that 83.1% of 337 PDAC cases had some degree of CLDN18.2 expression, with 26.7% meeting the moderate-to-high expression threshold (≥75% of tumor cells with 2+/3+ staining) used in gastric cancer clinical trials (238). A separate study of 201 PDAC patients using the Ventana CLDN18 (43) CE IVD Assay reported positive CLDN18.2 expression in 27% of tumors (239). Importantly, high concordance of CLDN18.2 status between primary tumors and metastatic sites has been demonstrated, with correspondence rates of 70–100% depending on metastatic location (238, 240). CLDN18.2 expression was significantly lower in poorly differentiated tumors (G3 vs. G1/G2: OR = 0.37) and was associated with the classical molecular subtype rather than the basal-like subtype (237, 241).

Preclinical studies have demonstrated potent activity of CLDN18.2-directed bispecific antibodies in pancreatic cancer models. AMG 910, a CLDN18.2-targeted half-life extended BiTE molecule, induced potent cytotoxicity in PDAC cell lines and suppressed tumor growth in orthotopic pancreatic cancer models, with enhanced efficacy when combined with PD-1 inhibition (144). Notably, BiTE treatment converted regulatory T-cell function from immunosuppressive to immune-enhancing in syngeneic KPC models, a finding with particular relevance to the Treg-enriched PDAC microenvironment (134). Zhu et al. similarly demonstrated that CLDN18.2-targeting CD3 bispecific and diabody constructs inhibited tumor growth in pancreatic patient-derived xenograft models without obvious gastric toxicity (145).

Spevatamig (PT886), a CLDN18.2/CD47 bispecific antibody with an optimized anti-CD47 arm designed to bind CD47 more highly on cancer cells than on human red blood cells, represents a novel approach combining CLDN18.2 targeting with innate immune checkpoint blockade. In the TWINPEAK phase 1/2 study (NCT05482893), spevatamig combined with gemcitabine plus nab-paclitaxel in first-line metastatic PDAC demonstrated an ORR of 48%, DCR of 90%, median PFS of 7.3 months, and median OS exceeding 13 months (still maturing) at the 2 mg/kg QW dose level (N = 22), with no significant additive toxicity compared with chemotherapy alone and no grade ≥3 nausea or vomiting events (242, 243). Antitumor activity was demonstrated irrespective of tumor CLDN18.2 expression levels and RAS mutational status, suggesting that the CD47-targeting arm may enable activity against low CLDN18.2-expressing cells (243).

CEA-directed bispecific antibodies are also relevant to pancreatic cancer, as CEA is expressed in a substantial proportion of PDAC tumors. Cibisatamab and related CEA×CD3 constructs have been evaluated in CEA-positive solid tumors including pancreatic cancer, although clinical data specific to PDAC remain limited. B7-H3-directed bispecific antibodies, including the optimized B7-H3×CD3 construct CC-3, have demonstrated potent T-cell activation and tumor-cell killing in pancreatic cancer models with reduced cytokine release through CD3 affinity tuning (156).

Additional emerging approaches include ErbB-family-targeting bispecific antibodies. Rabia et al. generated bispecific antibodies against EGFR, HER2, and HER3 using the BiXAb platform and demonstrated deep antibody penetration and robust tumor growth reduction in preclinical mouse models of pancreatic cancer (244). Long et al. developed heterodimeric EGFRxHER2 T-cell-engaging bispecific antibodies that demonstrated potent anti-tumor effects in EGFR+HER2+ double-positive PDAC cell-line-derived and patient-derived xenograft models, with the potential to spare single-positive normal tissue (245). A PD-L1/CXCR4 bispecific nanobody also demonstrated superior anti-tumor efficacy compared with atezolizumab in a pancreatic cancer xenograft model by simultaneously blocking PD-L1 and inhibiting CXCL12-induced immune-cell migration (246). TIGIT-directed bispecific antibodies anchored to CDCP1 for tumor-localized immune activation have also shown enhanced NK cell-mediated cytotoxicity in PDAC models (247).

### 7.4 Stromal modulation strategies

The dense desmoplastic stroma of PDAC, which can constitute up to 85% of tumor bulk, represents one of the most formidable barriers to effective immunotherapy (223). Bispecific antibodies designed to modulate the tumor stroma are therefore of particular interest in this disease.

FAP-targeted bispecific antibodies represent the most advanced stromal modulation strategy. FAP-CD40, a bispecific antibody that induces CD40 stimulation solely in the presence of fibroblast activation protein, demonstrated potent, strictly FAP-dependent immune activation in preclinical models. Unlike nontargeted CD40 agonists, FAP-CD40 mediated complete regression of tumors, including KPC-4662 pancreatic cancer models, with preferential intratumoral accumulation and predominantly intratumoral immune activation while avoiding the systemic toxicity associated with conventional CD40 agonists (248). More recently, the combination of FAP-CD40 with PD1-IL2v was shown to reprogram immunologically cold KPC pancreatic tumors by driving *de novo* intratumoral T cell-dendritic cell clusters, requiring both CD4+ and CD8+ T cells for tumor regression (249).

FAP-4-1BBL (RO7122290), a fibroblast activation protein-targeted 4-1BB agonist, was designed to provide localized T-cell co-stimulation within FAP-expressing tumor stroma while minimizing systemic immune activation. This agent has demonstrated increased intratumoral CD8+ T-cell infiltration and encouraging translational activity in combination with cibisatamab in MSS colorectal cancer (70). Similar stromal modulation strategies are being explored in PDAC, where FAP-positive cancer-associated fibroblasts play a central role in immune exclusion (250, 251).

The combination of T-cell engagement with stromal co-stimulation is particularly attractive in PDAC because of the dual challenge of limited T-cell infiltration and active stromal suppression. Preclinical studies have demonstrated that combining mesothelin-directed T-cell engagers with PD-L1/4-1BB bispecific antibodies significantly enhanced tumor suppression in pancreatic cancer xenograft models compared with either agent alone (234). These findings support the development of multi-bispecific combination strategies designed to simultaneously redirect T cells, overcome stromal barriers, and provide localized co-stimulatory signaling within the PDAC microenvironment.

### 7.5 Clinical evidence and ongoing trials

Despite growing interest in bispecific antibodies for pancreatic cancer, clinical development remains at a relatively early stage compared with colorectal, gastric, and hepatocellular carcinomas. This slower progress reflects the unique biological challenges posed by PDAC, including profound immune suppression, extensive stromal desmoplasia, and limited baseline T-cell infiltration. Consequently, most bispecific antibody programs remain in phase I evaluation or preclinical development, although several platforms have generated encouraging early signals of activity.

The development of bispecific antibodies in PDAC faces several challenges that are less prominent in other gastrointestinal malignancies. The dense desmoplastic and immunosuppressive tumor microenvironment may limit the efficacy of T-cell-engaging constructs even when immune cells are successfully redirected toward tumor cells. Mesothelin-directed therapies face the additional challenge of antigen shedding, although membrane-restricted targeting strategies such as JNJ-79032421 appear capable of overcoming this limitation (27, 28). Similarly, although CLDN18.2 is expressed in a substantial proportion of pancreatic cancers, expression levels are generally lower and more heterogeneous than those observed in gastric cancer, creating uncertainty regarding optimal biomarker thresholds and patient selection strategies (237, 238). The early clinical experience with spevatamig provides an important proof-of-concept that bispecific antibodies can generate meaningful activity in PDAC. The incorporation of CD47 blockade may broaden activity beyond tumors with high CLDN18.2 expression by recruiting innate immune effector mechanisms, potentially addressing one of the major limitations of conventional antigen-restricted approaches (242, 243). However, it remains unclear whether these encouraging early results will translate into durable benefit in larger studies. In addition, the rapid clinical deterioration and limited functional reserve frequently seen in advanced PDAC may complicate the implementation of step-up dosing schedules and prolonged treatment strategies commonly used for T-cell-engaging therapies.

Overall, the pancreatic cancer bispecific antibody landscape is evolving rapidly and encompasses a broad range of targets, including mesothelin, CLDN18.2, CEA, B7-H3, ErbB-family receptors, and stromal components. The increasing integration of T-cell engagement, innate immune activation, stromal remodeling, and co-stimulatory signaling reflects a growing recognition that no single mechanism is likely to overcome the complex immune resistance of PDAC. While most programs remain in early development, the diversity of current strategies and the encouraging preliminary results observed with agents such as spevatamig suggest that bispecific antibodies may ultimately play an important role in expanding the therapeutic options available for this historically immunotherapy-refractory disease.

## 8 Bispecific antibodies in biliary tract cancer

### 8.1 Current role of immunotherapy in biliary tract cancer

Biliary tract cancers (BTCs), including intrahepatic cholangiocarcinoma, extrahepatic cholangiocarcinoma, and gallbladder carcinoma, are aggressive malignancies with rising global incidence and poor long-term survival (252). The phase III TOPAZ-1 trial established durvalumab plus gemcitabine and cisplatin (GemCis) as a first-line standard, and KEYNOTE-966 subsequently confirmed the benefit of adding pembrolizumab to GemCis (253, 254). Current NCCN guidelines list GemCis plus either durvalumab or pembrolizumab as preferred first-line regimens (255). Second-line options remain limited, with FOLFOX producing low response rates and a median OS of approximately 6–9 months (256, 257).

Molecular profiling has expanded the therapeutic landscape by identifying actionable alterations including FGFR2 fusions, IDH1 mutations, BRAF V600E mutations, HER2 overexpression, MSI-H/dMMR, NRG1 fusions, RET fusions, and KRAS G12C mutations (255). However, single-agent PD-1 or PD-L1 inhibitors have shown only limited activity in unselected BTC populations (ORR 3–11% outside MSI-H/dMMR tumors), and the immunosuppressive tumor microenvironment, including dense desmoplastic stroma and limited cytotoxic T-cell infiltration, restricts the activity of checkpoint blockade (257, 258). These limitations have created strong interest in bispecific antibodies targeting HER2, PD-1/CTLA-4, PD-1/VEGF, DLL4/VEGF-A, CLDN18.2, FGFR2, and CDH17 pathways.

### 8.2 Zanidatamab: the first approved bispecific antibody in biliary tract cancer

Zanidatamab is a biparatopic HER2-directed bispecific antibody that simultaneously binds two distinct extracellular domains of HER2, corresponding to the trastuzumab and pertuzumab binding regions. This dual engagement promotes HER2 clustering, receptor internalization and degradation, inhibition of HER2 homo- and heterodimerization, and immune-mediated cytotoxicity through ADCC, ADCP, and CDC (256). In November 2024, zanidatamab received accelerated FDA approval for adults with previously treated, unresectable or metastatic HER2-positive BTC, followed by conditional approval from the EMA and NMPA in 2025 (259).

The pivotal HERIZON-BTC-01 trial enrolled 80 patients with HER2-amplified BTC previously treated with gemcitabine-based therapy. At final analysis, with a median follow-up of 33.4 months, zanidatamab achieved a confirmed objective response rate of 41.3%, including 3 complete responses and 30 partial responses (260). Median response duration was 14.9 months, and median overall survival was 15.5 months (260). Importantly, benefit was strongly dependent on HER2 expression level. Among patients with IHC 3+ tumors, the confirmed response rate was 51.6%, and median overall survival was 18.1 months, compared with an objective response rate of only 5.6% and median overall survival of 5.2 months in IHC 2+ disease (260). A *post hoc* landmark analysis further showed that objective response was associated with longer survival, with post-landmark median overall survival of 24.5 months among week-9 responders compared with 8.9 months for all others (261).

Real-world data have supported the clinical relevance of these findings. In a French multicenter retrospective cohort of 20 patients, zanidatamab produced a confirmed partial response rate of 40%, median progression-free survival of 6.7 months, and a 1-year overall survival rate of 79.1%, with better outcomes in IHC 3+ compared with IHC 2+ tumors (262). An external control analysis comparing zanidatamab with real-world second-line chemotherapy also suggested a substantial survival advantage, with median overall survival of 18.07 versus 3.29 months (263).

Zanidatamab has generally shown a manageable safety profile. The most common treatment-related adverse events were diarrhea and infusion-related reactions, while grade ≥3 treatment-related adverse events occurred in 21% of patients and no treatment-related deaths were reported (260, 264). The ongoing phase III HERIZON-BTC-302 trial is evaluating zanidatamab in combination with first-line standard-of-care therapy for HER2-positive BTC (260).

### 8.3 Bintrafusp alfa: lessons from PD-L1/TGF-β dual targeting

Bintrafusp alfa, a bifunctional fusion protein combining PD-L1 blockade with TGF-β sequestration, was among the earliest bispecific-like agents evaluated in BTC. In a phase I expansion cohort of 30 pretreated Asian patients, bintrafusp alfa monotherapy achieved an objective response rate of 20%, with durable responses and median overall survival of 12.7 months. Activity was observed regardless of PD-L1 expression or MSI status (265).

However, subsequent larger studies produced disappointing results. In a phase II study of 159 patients with chemorefractory BTC, bintrafusp alfa monotherapy achieved an objective response rate of only 10.7%, with median progression-free survival of 1.8 months and median overall survival of 7.6 months, failing to meet its prespecified primary endpoint (265). More importantly, a randomized phase II/III trial comparing bintrafusp alfa plus GemCis with GemCis alone in the first-line setting showed no survival benefit, with median overall survival of 11.5 months in both arms. The combination was also associated with increased bleeding events and early deaths, leading to early termination of the study (266).

The bintrafusp alfa experience highlights an important lesson for BTC drug development: biologically rational dual-pathway inhibition does not necessarily translate into clinical benefit without appropriate patient selection. The negative first-line trial may reflect the complex and context-dependent role of TGF-β signaling in BTC, as well as the lack of predictive biomarkers capable of identify patients most likely to benefit (267). A meta-analysis across gastrointestinal tumors similarly suggested modest overall efficacy for bintrafusp alfa, with pooled objective response rates below 20% (268).

### 8.4 Dual checkpoint and anti-angiogenic bispecific antibodies

Dual checkpoint bispecific antibodies are now being explored as a strategy to improve immune activation in BTC. Vudalimab, a PD-1/CTLA-4 bispecific antibody, is currently under evaluation in previously treated advanced BTC, building on earlier solid tumor data showing preliminary antitumor activity (269).

Cadonilimab, another PD-1/CTLA-4 bispecific antibody, has generated particularly encouraging early data. In the phase II BicureX trial, cadonilimab plus GemCis as first-line treatment for advanced BTC achieved an objective response rate of 59.3%, disease control rate of 88.1%, and median progression-free survival of 9.0 months, with two complete responses (270). Notably, all evaluable patients were microsatellite-stable and had low median tumor mutational burden, suggesting activity beyond conventional immunotherapy-sensitive biomarkers. Grade ≥3 treatment-related adverse events occurred in 70% of patients, mainly hematologic toxicities, and two cases of grade 4 immune-related myocarditis were reported (270). Cadonilimab is also being investigated as conversion therapy for unresectable BTC, with early data showing an objective response rate of 54.5% and an R0 conversion rate of 18.2% in treatment-naive patients (271). In later-line therapy, cadonilimab combined with liposomal irinotecan, fluorouracil, and leucovorin demonstrated an objective response rate of 29.4% and median progression-free survival of 4.7 months, including activity in patients previously treated with checkpoint inhibitors (272).

Anti-angiogenic bispecific antibodies represent another promising approach. Ivonescimab, a PD-1/VEGF bispecific antibody, has shown encouraging activity in first-line BTC. In a phase II study of 22 patients treated with ivonescimab plus GemCis, the objective response rate was 63.6%, the disease control rate was 100%, median progression-free survival was 8.5 months, and median overall survival was 16.8 months, with activity observed regardless of PD-L1 status (273). A subsequent study combining ivonescimab with GemCis and stereotactic body radiotherapy reported an objective response rate of 55.0% and disease control rate of 90.0% in advanced BTC (274).

### 8.5 DLL4/VEGF-a bispecific antibodies

Tovecimig (CTX-009) is a DLL4/VEGF-A bispecific antibody that represents a mechanistically distinct anti-angiogenic strategy. DLL4/Notch signaling has been implicated in cholangiocarcinogenesis, while VEGF-A overexpression is frequently observed in cholangiocarcinoma and is associated with metastasis and poor prognosis (275, 276). By simultaneously blocking DLL4-Notch and VEGF-A-VEGFR signaling, tovecimig targets both VEGF-dependent and VEGF-independent angiogenic pathways.

In a phase II study of patients with advanced BTC previously treated with one or two lines of therapy, tovecimig plus paclitaxel achieved an objective response rate of 37.5%, exceeding historical response rates for standard second-line therapy (275). Based on this signal, the randomized phase II/III COMPANION-002 trial is evaluating tovecimig plus paclitaxel versus paclitaxel alone as second-line therapy for advanced BTC, with objective response rate as the primary endpoint and crossover permitted (275, 277).

### 8.6 Emerging targets: CLDN18.2, FGFR2, and CDH17

Several additional targets may further expand the role of bispecific antibodies in BTC. CLDN18.2 expression has been identified in a subset of BTCs, although prevalence varies substantially by anatomic subsite and scoring threshold. In a cohort of 237 BTCs, CLDN18 expression was detected in 29.5% of cases, with higher rates in gallbladder carcinoma and extrahepatic cholangiocarcinoma than in intrahepatic cholangiocarcinoma (278). However, using the stringent gastric cancer threshold of ≥75% tumor cells with moderate-to-strong staining, only 5.5% of BTCs were classified as positive (278). A German study using the VENTANA antibody reported CLDN18.2 positivity in 13.1% of cholangiocarcinomas, with the highest rates in perihilar CCA and large duct-type intrahepatic CCA (279). A pan-cancer analysis similarly reported CLDN18.2 expression in 21.7% of BTCs using a less stringent threshold (280). These findings suggest that CLDN18.2-directed therapies may be relevant to a selected subset of BTCs, particularly gallbladder carcinoma and perihilar CCA. Early data from the ZSAB-Calm study evaluating cadonilimab plus the CLDN18.2-targeting antibody-drug conjugate LM-302 reported a best overall response of 50% in dose escalation (281). FGFR2 biparatopic antibodies represent another innovative strategy, particularly for FGFR2 fusion-driven cholangiocarcinoma. Chaturantabut et al. generated biparatopic antibodies targeting distinct extracellular FGFR2 epitopes and identified constructs capable of blocking FGFR2 fusion-driven signaling and malignant growth (282). These antibodies demonstrated *in vivo* efficacy, synergy with FGFR kinase inhibitors, and activity against FGFR2 fusions harboring kinase-domain resistance mutations, suggesting potential value after resistance to approved FGFR inhibitors (282).

CDH17 is another emerging antigen of interest in gastrointestinal malignancies, including biliopancreatic cancers. CDH17 is aberrantly expressed in a substantial proportion of biliopancreatic adenocarcinomas, while normal expression is largely restricted to tight junctions between mucosal epithelial cells, potentially limiting immune accessibility (283, 284). Cabotamig (ARB202), a CDH17×CD3 bispecific T-cell engager, is currently in first-in-human evaluation in CDH17-expressing gastrointestinal cancers, including cholangiocarcinoma. Early data suggest dose-responsive biomarker activity, manageable CRS, and no persistent gastrointestinal toxicity (285). BI 905711, a tetravalent bispecific antibody targeting TRAILR2 and CDH17, demonstrated potent and selective apoptosis induction in CDH17-positive tumor cells, with marked reduction in activity against CDH17-negative cells and single-agent tumor regressions in preclinical models (286).

### 8.7 Clinical evidence and ongoing trials

Biliary tract cancer is notable as the first gastrointestinal malignancy in which a bispecific antibody, zanidatamab, has received regulatory approval. The robust activity of zanidatamab in HER2 IHC 3+ BTC, together with emerging data for cadonilimab, ivonescimab, and tovecimig, suggests that bispecific antibodies may become increasingly relevant across both biomarker-selected and broader BTC populations.

However, several challenges remain. HER2 expression in BTC is heterogeneous and varies substantially by anatomic subtype, with the highest prevalence generally observed in gallbladder carcinoma and extrahepatic cholangiocarcinoma (255). CLDN18.2 expression is also variable and generally less frequent than in gastric cancer when stringent scoring thresholds are applied. In addition, the optimal sequencing of bispecific antibodies with chemoimmunotherapy, FGFR inhibitors, IDH1 inhibitors, antibody-drug conjugates, and other targeted therapies remains undefined. The dense stromal and immunosuppressive microenvironment characteristic of cholangiocarcinoma may further limit the efficacy of T-cell-engaging strategies, emphasizing the need for rational combinations that address both immune redirection and stromal resistance.

Overall, the BTC bispecific antibody pipeline is rapidly diversifying. Current strategies include HER2 biparatopic antibodies, dual checkpoint blockades, PD-1/VEGF combinations, DLL4/VEGF-A dual inhibition, FGFR2 biparatopic antibodies, CLDN18.2-directed approaches, and CDH17-targeted T-cell engagers. The transition from the first regulatory approval to multiple ongoing phase II and III trials marks an important inflection point in the development of bispecific antibodies for biliary tract cancer.

## 9 Bispecific antibodies in esophageal cancer and gastrointestinal neuroendocrine neoplasms

### 9.1 Current role of immunotherapy in esophageal cancer

Esophageal cancer is the seventh most common malignancy worldwide, with esophageal squamous cell carcinoma (ESCC) and esophageal adenocarcinoma (EAC) representing biologically distinct diseases (288, 289). PD-1/PD-L1 blockade combined with chemotherapy has established a new standard of care in ESCC, based on the phase III KEYNOTE-590, CheckMate-648, and RATIONALE-306 trials (290–292). A meta-analysis of seven randomized trials demonstrated significant improvements in both OS (HR 0.68) and PFS (HR 0.62) with ICI-based chemoimmunotherapy, although the survival advantage was largely driven by ESCC rather than EAC (293). Current NCCN guidelines recommend fluoropyrimidine- and platinum-based chemotherapy combined with nivolumab, pembrolizumab, or tislelizumab as preferred first-line regimens for ESCC and for HER2-negative esophageal or EGJ adenocarcinoma with PD-L1 CPS ≥1 (294). In the adjuvant setting, nivolumab following neoadjuvant chemoradiotherapy and R0 resection remains a category 1 recommendation based on CheckMate-577.

Despite these advances, most patients ultimately experience disease progression, and patients with low PD-L1 expression or liver metastases derive less benefit. These limitations have stimulated the development of bispecific antibodies, with dual checkpoint inhibition (PD-1/CTLA-4, PD-L1/CTLA-4), combined checkpoint and anti-angiogenic blockade (PD-1/VEGF), and tumor-targeting strategies (EGFR/HER3, EGFR/B7-H3) representing the principal approaches under investigation.

### 9.2 Dual checkpoint bispecific antibodies in esophageal cancer

Dual checkpoint bispecific antibodies currently represent the most advanced bispecific platform in esophageal cancer. Unlike T-cell-engaging constructs that redirect cytotoxic lymphocytes toward tumor-associated antigens, these agents simultaneously inhibit multiple immune checkpoint pathways to augment endogenous antitumor immunity.

The phase III SKYSCRAPER-08 trial evaluated tiragolumab (anti-TIGIT) plus atezolizumab (anti-PD-L1) combined with chemotherapy versus chemotherapy alone in 461 patients with treatment-naïve unresectable ESCC (295). Median PFS improved from 5.4 to 6.2 months (HR 0.56; 95% CI 0.45–0.70; p<0.0001), while median OS improved from 11.1 to 15.7 months (HR 0.70; 95% CI 0.55–0.88; p = 0.0024). Although tiragolumab and atezolizumab are administered as separate monoclonal antibodies rather than as a single bispecific construct, these findings provide compelling clinical validation for simultaneous TIGIT and PD-L1 blockade. Earlier data from the MORPHEUS-EC study similarly demonstrated improved response rates with the addition of TIGIT inhibition, with ORRs of 67.7%, 53.8%, and 47.8% for the triplet regimen, atezolizumab plus chemotherapy, and chemotherapy alone, respectively (296).

Cadonilimab, a PD-1/CTLA-4 bispecific antibody, has generated some of the most promising clinical data among true bispecific checkpoint inhibitors in ESCC. In the COMPASSION-03 phase 1b/2 trial, cadonilimab monotherapy achieved an ORR of 18.2%, with a median PFS of 3.5 months and median OS of 9.4 months in previously treated patients (64). More encouraging results were observed in the first-line setting, where cadonilimab combined with taxane and cisplatin achieved an ORR of 81.4%, a DCR of 97.7%, and a median PFS of 7.10 months (297). Interestingly, specific DNA methylation signatures were associated with clinical response, suggesting a potential role for epigenetic biomarkers in patient selection (297). Cadonilimab is also being investigated in neoadjuvant treatment strategies, including combinations with chemoradiotherapy and chemotherapy-free regimens incorporating antiangiogenic therapy (298).

Another dual checkpoint approach is represented by KN046, a PD-L1/CTLA-4 bispecific antibody. In a phase Ib study combining KN046 with chemotherapy and palliative radiotherapy in advanced ESCC, the regimen achieved an ORR of 41.7%, median PFS of 7.8 months, and median OS of 15.9 months with manageable toxicity (299). Collectively, these findings support the growing role of dual checkpoint inhibition as a promising strategy for overcoming resistance to conventional PD-1-directed therapy.

SHR-1701, a bifunctional anti-PD-L1/TGF-βRII fusion protein, has further expanded this concept by simultaneously targeting immune checkpoint signaling and TGF-β-mediated immune suppression. When combined with neoadjuvant chemoradiotherapy in resectable locally advanced ESCC, SHR-1701 achieved a pCR rate of 30.0% and a 100% R0 resection rate, highlighting the potential of combined immunomodulatory approaches in earlier-stage disease (300).

### 9.3 PD-1/VEGF bispecific antibodies and other emerging strategies

Beyond dual checkpoint blockade, bispecific antibodies integrating immune checkpoint inhibition with antiangiogenic activity are emerging as a promising therapeutic strategy. Ivonescimab, a PD-1/VEGF bispecific antibody, is currently being explored across multiple upper gastrointestinal malignancies. Although most available data derive from gastric and gastroesophageal junction adenocarcinoma, combination therapy with ivonescimab, cadonilimab, and chemotherapy achieved an ORR of 71.7% and a DCR of 95.7%, providing proof-of-concept for simultaneous immune and angiogenic pathway inhibition (Wang et al., 2026 ASCO).

Bispecific targeting strategies have also expanded into antibody-drug conjugate platforms. BL-B01D1, a first-in-class EGFR/HER3 bispecific ADC, demonstrated encouraging activity in previously treated metastatic ESCC, achieving a confirmed ORR of 39.6% at the recommended phase II dose and a DCR of 79.2% (300). These results have supported progression into phase III clinical testing.

Similarly, IBI334, a tumor-selective EGFR/B7-H3 bispecific antibody, demonstrated enhanced EGFR blockade and receptor degradation compared with conventional EGFR-directed antibodies while minimizing off-target toxicity (301). The favorable preclinical safety profile and ongoing phase I evaluation highlight the potential of tumor-selective bispecific platforms to expand therapeutic windows in esophageal cancer.

### 9.4 Bispecific antibodies in gastrointestinal neuroendocrine neoplasms

Gastrointestinal neuroendocrine neoplasms (GI-NENs), particularly poorly differentiated gastroenteropancreatic neuroendocrine carcinomas (GEP-NECs), remain difficult-to-treat malignancies with limited therapeutic options following platinum-based chemotherapy failure (302). Among emerging targets, DLL3 has attracted considerable attention because of its frequent overexpression in NECs and minimal expression in normal tissues.

Obrixtamig (BI 764532), a DLL3/CD3 IgG-like T-cell engager, has emerged as the most clinically advanced bispecific antibody in this setting. In an ongoing phase I study, patients with DLL3-high extrapulmonary NECs achieved an ORR of 40.0%, with particularly encouraging activity observed in GEP-NECs, where response rates reached 50.0% (303). Responses were durable, with a median duration of response of 7.9 months, and the safety profile remained manageable. These findings have supported progression into the phase II DAREON-5 study (304).

Alveltamig (ZG006), a trispecific DLL3/DLL3/CD3 T-cell engager, has also demonstrated substantial activity in refractory NECs. In a randomized phase II study, patients with DLL3-high tumors achieved an ORR of 56.3% and a median PFS of 8.41 months, with responses observed across multiple GI primary sites, including gastric, gallbladder, rectal, and colorectal NECs (305). Cytokine release syndrome was largely low grade, supporting the feasibility of this approach.

Additional DLL3-targeted strategies are rapidly advancing. Peluntamig (PT217), a DLL3/CD47 bispecific antibody, is currently being evaluated in both monotherapy and combination regimens, while tarlatamab, already approved for small-cell lung cancer, is being investigated in basket trials enrolling DLL3-positive tumors regardless of tissue origin (266, 306).

Biomarker development is emerging as a critical component of DLL3-directed therapy. Circulating tumor cell-based DLL3 quantification has demonstrated strong predictive value for treatment response and may provide a noninvasive method for patient selection and disease monitoring (307).

### 9.5 Clinical evidence and ongoing trials

Bispecific antibody development in esophageal cancer is currently dominated by dual checkpoint strategies, reflecting the established role of PD-1/PD-L1 blockade and the biological rationale for simultaneously targeting complementary immune inhibitory pathways. Among these agents, cadonilimab has generated the most mature clinical evidence, particularly in combination with chemotherapy in the first-line setting. The positive results of SKYSCRAPER-08 further validate the concept of dual immune checkpoint inhibition in ESCC, even though TIGIT and PD-L1 blockade were achieved through separate monoclonal antibodies rather than a single bispecific construct. In contrast, tumor-directed bispecific platforms remain at an earlier stage of development, although EGFR-targeting agents such as IBI334 and BL-B01D1 are beginning to show promising activity.

In GI neuroendocrine neoplasms, DLL3-directed bispecific antibodies may represent a major therapeutic advance for a disease with historically poor outcomes and limited treatment options. The response rates achieved with obrixtamig and alveltamig substantially exceed historical outcomes observed with conventional second-line therapies and rank among the most encouraging efficacy signals reported in GEP-NECs. Importantly, the strong association between DLL3 expression and clinical response highlights the critical role of biomarker-guided patient selection. As DLL3-targeted therapies advance into later-phase studies, standardization of DLL3 testing and integration of liquid biopsy-based biomarkers will likely become essential components of clinical implementation.

## 10 Future directions

Next-generation engineering platforms are expected to play a central role in improving the therapeutic index of bispecific antibodies. Protease-activatable masked constructs, conditional co-stimulatory antibodies, Fc-engineered molecules, and tumor-selective activation platforms are being developed to restrict immune activation to the tumor microenvironment and minimize systemic toxicity (189, 309). Trispecific and multifunctional immune engagers are also entering clinical evaluation to simultaneously target tumor antigens, immune checkpoints, and co-stimulatory pathways within a single molecule (23, 90). AI-assisted modeling is increasingly being applied to antibody design, structural prediction, affinity optimization, and rational selection of dual-targeting strategies (309, 310).

The target landscape is also expanding. Current development remains concentrated around validated targets such as PD-1, CTLA-4, EGFR, HER2, and CLDN18.2; newer combinations are integrating checkpoint blockades with stromal, angiogenic, innate immune, and co-stimulatory pathways. Examples include PD-L1×TGF-β, PD-1×4-1BB, CD47×PD-L1, and VEGF×TGF-β platforms (10). Novel tumor-associated antigens such as CDH17, LY6G6D, and DLL3 are broadening the targetable space across GI malignancies. *In situ* production of bispecific antibodies through oncolytic viruses or adeno-associated viral vectors may offer another strategy to overcome biodistribution and penetration limitations in solid tumors (189).

Integration with cellular therapies is another promising direction. Bispecific antibodies may be paired with CAR-T cells, γδ T cells, or NK cell-based therapies to improve effector-cell trafficking, activation, and persistence within solid tumors (14, 189). In this context, bispecific antibodies could function not only as direct therapeutics but also as bridging or sensitizing agents that remodel the tumor microenvironment and enhance the activity of adoptive cellular therapies.

Clinical trial design is evolving in parallel. Platform trials such as GEMINI-Gastric allow multiple bispecific and immune-modulating combinations to be evaluated within shared infrastructures, accelerating comparison across regimens and enabling more efficient biomarker-driven development. The integration of tissue-based testing, liquid biopsy monitoring, spatial transcriptomics, and AI-driven predictive modeling represents the most promising route toward personalized bispecific antibody therapy (10, 125).

Finally, bispecific antibodies offer practical advantages compared with several competing immunotherapeutic modalities. Unlike autologous CAR-T-cell therapy, bispecific antibodies are generally off-the-shelf products that can be administered without individualized cell manufacturing. Compared with antibody-drug conjugates, they avoid payload-related toxicities while enabling simultaneous immune engagement or pathway blockade. These features may support broader accessibility and outpatient administration, although cost, manufacturing scale, and healthcare-system capacity will remain important determinants of real-world adoption (105).

## 11 Conclusions

Bispecific antibodies have evolved from experimental immunotherapy platforms into one of the fastest-growing areas of clinical development in GI oncology. The field has moved beyond early-phase safety studies toward randomized trials across colorectal, gastric, biliary tract, pancreatic, hepatocellular, esophageal, and neuroendocrine cancers. A recent landscape analysis identified 681 registered clinical trials evaluating 183 bispecific antibodies in solid tumors, with GI cancers representing one of the major areas of investigation (10). Several milestones have already validated the clinical potential of this approach. The approval of zanidatamab for HER2-positive biliary tract cancer, the positive phase III results of HERIZON-GEA-01 in gastric cancer, the clinical validation of CLDN18.2-directed therapies, and the emerging activity of DLL3-directed T-cell engagers in neuroendocrine carcinomas collectively demonstrate that bispecific antibodies can generate meaningful activity across multiple GI malignancies (11). However, the full promise of bispecific antibodies will depend on solving several persistent challenges: overcoming stromal and immune exclusion, managing antigen heterogeneity and treatment-emergent target loss, reducing on-target/off-tumor toxicity, improving manufacturing scalability, and implementing robust biomarker-guided patient selection. If these barriers can be addressed through rational engineering, combination strategies, and biomarker-integrated clinical development, bispecific antibodies are likely to become a central component of next-generation precision immunotherapy in gastrointestinal oncology.
